# Geology and land use as key drivers for hydrogeochemistry in a mining district of the Quadrilátero Ferrífero, Brazil: implications for water management strategies

**DOI:** 10.1007/s10653-026-02989-0

**Published:** 2026-01-29

**Authors:** Gabriel Negreiros Salomão, Normara Yane Mar da Costa Andrade, Gabriel Soares de Almeida, Rafael Tarantino Amarante, Roberto Dall’Agnol, Paulo Rógenes Monteiro Pontes, Prafulla Kumar Sahoo, Lucas Pereira Leão, Eduardo Duarte Marques, Emmanoel Vieira da Silva-Filho

**Affiliations:** 1 Instituto Tecnológico Vale, Rua Boaventura da Silva, 955, Belém, Pará 66055-090 Brazil; 2 Instituto de Geociências, Universidade Federal Do Pará, Rua Augusto Corrêa, 01, Guamá, Belém, , Pará Brazil; 3 Department of Environmental Science and Technology, School of Environment and Earth Sciences, Central University of Punjab, VPO-Ghudda, Bathinda, Punjab 151401 India; 4 Departamento de Geologia, Campus Morro do Cruzeiro s/n—Bauxita, Universidade Federal de Ouro Preto (UFOP), Ouro Preto, Minas Gerais, Brazil; 5 Geological Survey of Brazil (SGB/CPRM), Av. Brasil, 1731, Funcionários, Belo Horizonte, Minas Gerais 30140-002 Brazil; 6 Instituto de Química, Universidade Federal Fluminense (UFF), Outeiro São João Baptista S/N, Centro, Niterói, Rio de Janeiro, 24020-141 Brazil

**Keywords:** Surface water, Geochemical background, Geochemical baseline, Water quality guideline, Congonhas mineral district

## Abstract

This study investigated the hydrogeochemical characteristics of surface waters in the Congonhas Mineral District (CMD), located in the southern portion of Quadrilátero Ferrífero, Brazil. A total of 38 sites were monitored between 2021 and 2024 to understand seasonal and spatial variability across distinct lithologies and land uses. Hydrogeochemical patterns revealed dominant mixed bicarbonate facies associated with metavolcano-sedimentary terrains, while domains of granitoids exhibited alkali enrichment. Waters under the influence of larger Urban settlements were enriched in Na, Cl, sulfate, and nutrients. By integrating geospatial classification, seasonal sampling, and robust statistical techniques, we investigated the behavior of Fe and Mn, key elements influenced by both natural geological sources and mining activities. Reference values for geochemical background and baseline thresholds, based on samples from preserved and mixed land use areas, respectively, were estimated using three distinct statistical approaches. Among these, the upper tolerance limit (UTL) method was considered the most consistent and suitable. Spatial and seasonal patterns revealed elevated Fe and Mn levels during the rainy season, particularly in areas influenced by mining and urbanization. The proposed reference values provide a realistic basis for identifying contamination, and can give support for more realistic regulatory frameworks, and definition of strategies for water quality management. The obtained results highlight the relevance of tailored guidelines in mining contexts, where reference values adopted by regulatory agencies may not reflect local geochemical conditions.

**Supplementary Information:**

The online version contains supplementary material available at 10.1007/s10653-026-02989-0.

## Introduction

Aquatic ecosystems are highly complex, subject to multiple natural and anthropogenic influences. Changes in water quality result from factors such as chemical weathering, biogeochemical cycles, effluent discharge, agricultural practices, and mining activities (Reimann & Garrett, 2005; Sahoo et al., 2019). Unregulated land use, along with urban growth and the expansion of industry and agriculture, has reduced the availability and quality of surface waters in many parts of the world (Allan, 2004; Dupasa et al., 2015; Menezes et al., 2014; Philippi Junior & Malheiros, 2005; Turner & Rabalais, 2003). Domestic and industrial effluents, along with intensive agriculture, introduce nutrients and heavy metals into water bodies, inducing eutrophication and reducing biodiversity (Alloway, 1997; Duffus, 2001; Ngoye & Machiwa, 2004).

Understanding the interactions between land use and land cover (LULC) and water quality is a key tool for developing effective environmental management strategies. Physical and chemical assessments of water, considering spatial and temporal variability, are essential for evaluating the effects of human activities on aquatic ecosystems (Christopher et al., 2010; Jones et al., 1999; Koçer & Sevgili, 2014; Limburg & Schmidt, 1990). Case studies have demonstrated these effects: 1) the deforestation associated to agricultural and livestock activities impairs water quality (Zhang et al., 2021); 2) high concentrations of phosphorus, ammoniacal nitrogen, and sulfate and increase of biochemical oxygen demand, are commonly observed in urbanized areas (Severo et al., 2021); and, 3) historical mining and farming have decreased the water quality of the Paraúna River in Minas Gerais, although it still serves a dilution function for pollutants in the Rio das Velhas (Nonato, 2007; Santos, 2017).

Establishing geochemical reference values in surface waters is essential for differentiating natural levels from those affected by human activities (Escher et al., 2015; Reimann & Garrett, 2005). The geochemical background typically indicates natural concentrations in minimally disturbed environments, shaped by long-term geological and biogeochemical processes (Hinsby et al., 2008; Shand et al., 2007). In contrast, the baseline incorporates both natural conditions and diffuse anthropogenic inputs, representing the current state of a specific area (Albanese et al., 2007; Salminen & Gregorauskien, 2000). Estimating these values requires detailed consideration of local environmental attributes, including lithology, geomorphology, soil characteristics, climate, biological activity, and land use (Caritat & Cooper, 2016; Chen & Lu, 2014; Gałuszka, 2007a, 2007b; Reimann & Garrett, 2005; Sahoo et al., 2019; Salomão et al., 2020; Wang et al., 2020).

On the other hand, guideline values are regulatory thresholds established to protect human health and aquatic ecosystems in freshwater systems, such as rivers, streams, and lakes (CCME, 2019; ANZECC & ARMCANZ, 2000). These values are typically generalist in nature, developed to apply uniformly across large areas without necessarily accounting for natural geochemical variability (Gałuszka, 2007b; Smedley & Kinniburgh, 2002). This limitation is particularly critical in large-area countries, such as Brazil, where geological, climatic, and land-use heterogeneities are pronounced, and the uniform application of regulatory threshold reference values may obscure natural geochemical baselines (Sahoo et al., 2019; Salomão et al., 2020).

Several studies have shown that relying on generic threshold values as references can lead to overestimation or underestimation of contamination levels and to misclassification of environmental quality, ultimately resulting in misguided risk assessments or remediation strategies (Caritat & Cooper, 2016; Gałuszka & Migaszewski, 2012; Reimann & Garrett, 2005). Therefore, establishing more realistic and regionally adjusted guideline values based on local background and baseline concentrations is a more reliable approach for evaluating environmental risk and conducting regulatory assessments.

The study area is located within the Congonhas Mineral District (CMD), in the southern portion of the Quadrilátero Ferrífero (QF), a globally recognized metallogenic province in southeastern Brazil with many active Fe and Mn mines and a long history of gold exploitation dating back to the seventeenth century. The QF is characterized by a complex geological framework that exerts strong control over surface water chemistry (Roeser & Roeser, 2010). It is also one of Brazil's most densely populated regions, where intense mining, agriculture, and urbanization coexist with ecologically sensitive catchments and environmentally protected areas (Medeiros Filho et al., 2025; Rodrigues et al., 2012).

A recent regional-scale study provided an integrated geochemical characterization of surface waters in the QF, identifying patterns in major and trace elements across diverse lithologies and land-use settings (Almeida et al., 2025). In this context, the present study provides a more detailed assessment of a strategically important area within the CMD, an area of significant social, economic, and environmental relevance. By integrating land use, geology, and robust hydrochemical data, this work aims to refine the understanding of natural and anthropogenic contributions to water quality and to establish robust locally realistic geochemical guideline values for monitoring and management purposes.

## Study area

**Fig. 1:**
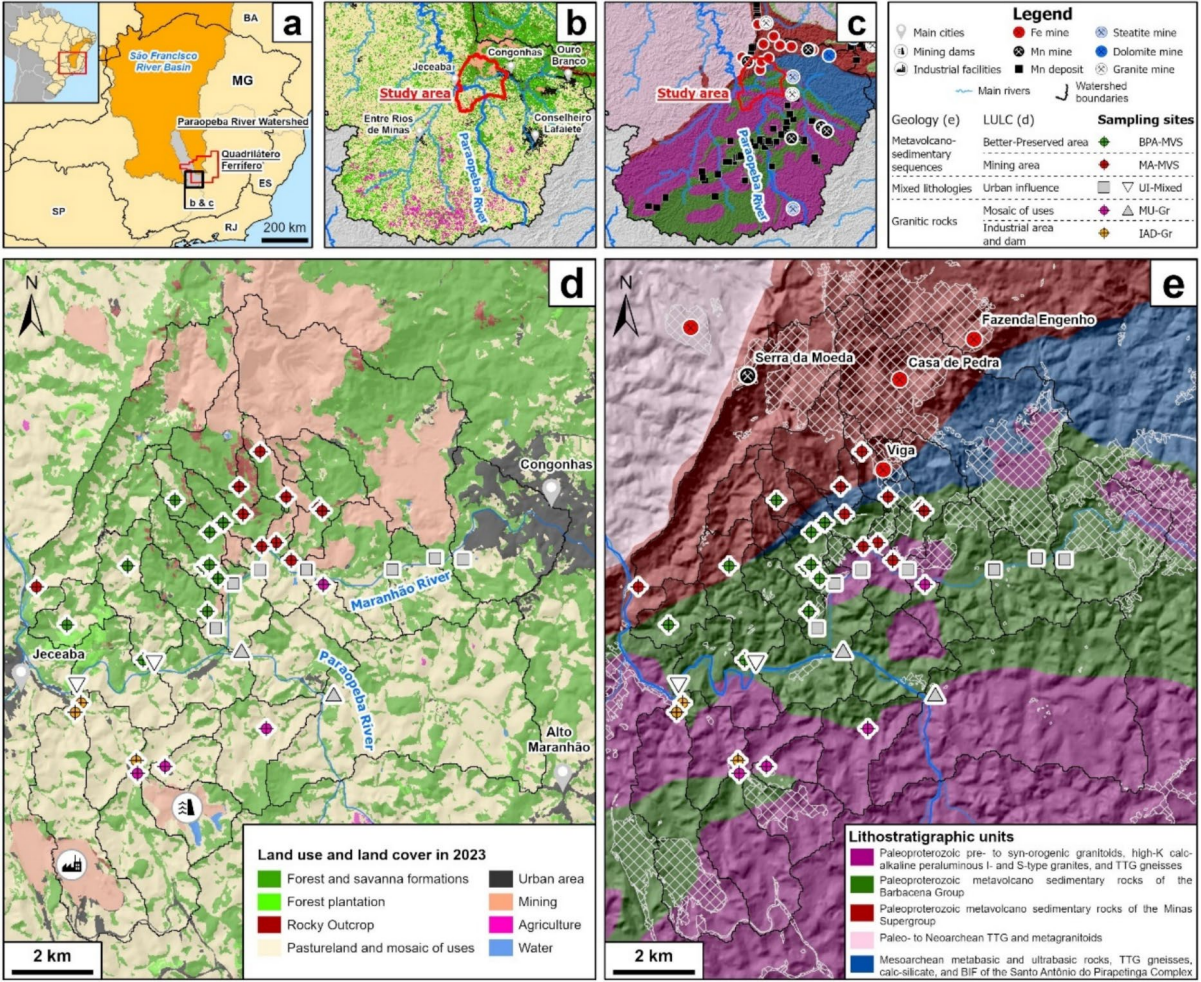
Location of the study area in the region of Congonhas Mineral District. **a** Regional context within the state of Minas Gerais, southeastern Brazil, showing the São Francisco River Basin, the Paraopeba River sub-basin, and the boundary of the Quadrilátero Ferrífero (QF). **b** Land use and land cover (LULC) and **c** geological maps of the Upper Paraopeba River basin, with the study area boundary highlighted. **d** Detailed LULC map of the study area in 2023. **e** Simplified geological map of the study area, showing surface water sampling points and the location of major iron mines (Viga, Casa de Pedra, and Engenho). More details about the stream flow direction are presented in Fig. 2. Source: Simplified layers of LULC and geology were retrieved from Mapbiomas (2024) and Silva et al. (2020), respectively

The study area is located in the QF region, south of Minas Gerais (MG) State (Fig. 1a), situated in the upper Paraopeba River basin (Fig. 1b, c). The local climate ranges from humid subtropical to high-altitude subtropical (Cwa and Cwb Köppen-Geiger types; Alvares et al., 2013), with an average annual precipitation of approximately 1450 mm (Nacional and de Meteorologi. (INMET). 2023). A well-defined seasonal pattern characterizes rainfall distribution, with a pronounced rainy season from November to April, which accounts for most of the annual precipitation, and a drier period from May to October (Fernandes & Moreira, 2022). The main urban center in the study area is Congonhas (Fig. 1d). This region is a major iron ore mining hub and encompasses a diverse range of land uses (Fig. 1b, d). In the northern sector, land use is dominated by mining activities, but remnants of natural formations (forest and savanna) and urban areas also play an important role in this sector. In contrast, the southern portion is primarily dominated by pasturelands interspersed with a complex mosaic of agricultural areas, grasslands, and minor occurrences of industrial areas and mining dams (Fig. 1d).

Geologically, the study area can be subdivided into two main domains (Fig. 1c, e): 1) Northern Domain, comprised of Mesoarchean to Paleoproterozoic metavolcano-sedimentary rocks, and 2) Southern Domain, corresponding to a Paleoproterozoic granitic terrain. The northern region is dominated by the Paleoproterozoic metavolcano-sedimentary sequences of the Barbacena group and Minas supergroup with subordinate occurrences of rocks of the Santo Antônio do Pirapetinga Complex, which is composed of Mesoarchean metamafic-ultramafic rocks, with intercalations of gneisses, calc-silicatic rocks, and banded iron formations (BIF) (Endo et al., 2019; Oliveira et al., 2023; Raposo, 1991). However, the dominant geological but a great influence comes from the Paleoproterozoic metavolcano-sedimentary sequences of the Barbacena Group and the Minas Supergroup.

The Barbacena Group consists of intercalated mafic/ultramafic rocks and igneous-metamorphic complexes, including phyllites, schists, quartzites, banded iron formations (BIF), and carbonate lenses (Cabral et al., 2019; Endo et al., 2019). Due to its metamorphic character and interaction with hydrothermal fluids, it created environments conducive to the mobilization and accumulation of Fe, Cu, Mn, and Au (Cabral et al., 2019; Endo et al., 2019). The Minas Supergroup comprises phyllites, Mn-rich schists, quartzites, itabirites, and dolomites (Alkmim & Marshak, 1998; Alkmim & Teixeira, 2017; Araújo et al., 2020; Rosière et al., 2008). This supergroup represents one of the most economically important stratigraphic units in the region, hosting the main Fe-ore deposits and acting as a significant geochemical source of Mn and other potentially toxic elements to the environment (Rossi, 2014).

The Southern Domain is dominated by granitic complexes, including subalkaline granites of the Alto Maranhão Suite and other Paleoproterozoic granitoids, important sources of Al, Ba, and Sr (Geológico and do Brasil. (CPRM). 2003; Martins, 2008). This lithological association comprises different Paleoproterozoic orthogneiss types (e.g., trondhjemitic, granodioritic, granitic), undeformed igneous bodies (e.g., gabbro, diorite, granite), and supracrustal volcano-sedimentary sequences locally enriched in Mn (Alkmim & Teixeira, 2017; Araújo et al., 2020).

## Materials and methods

### Sampling network design and analytical methods

**Fig. 2:**
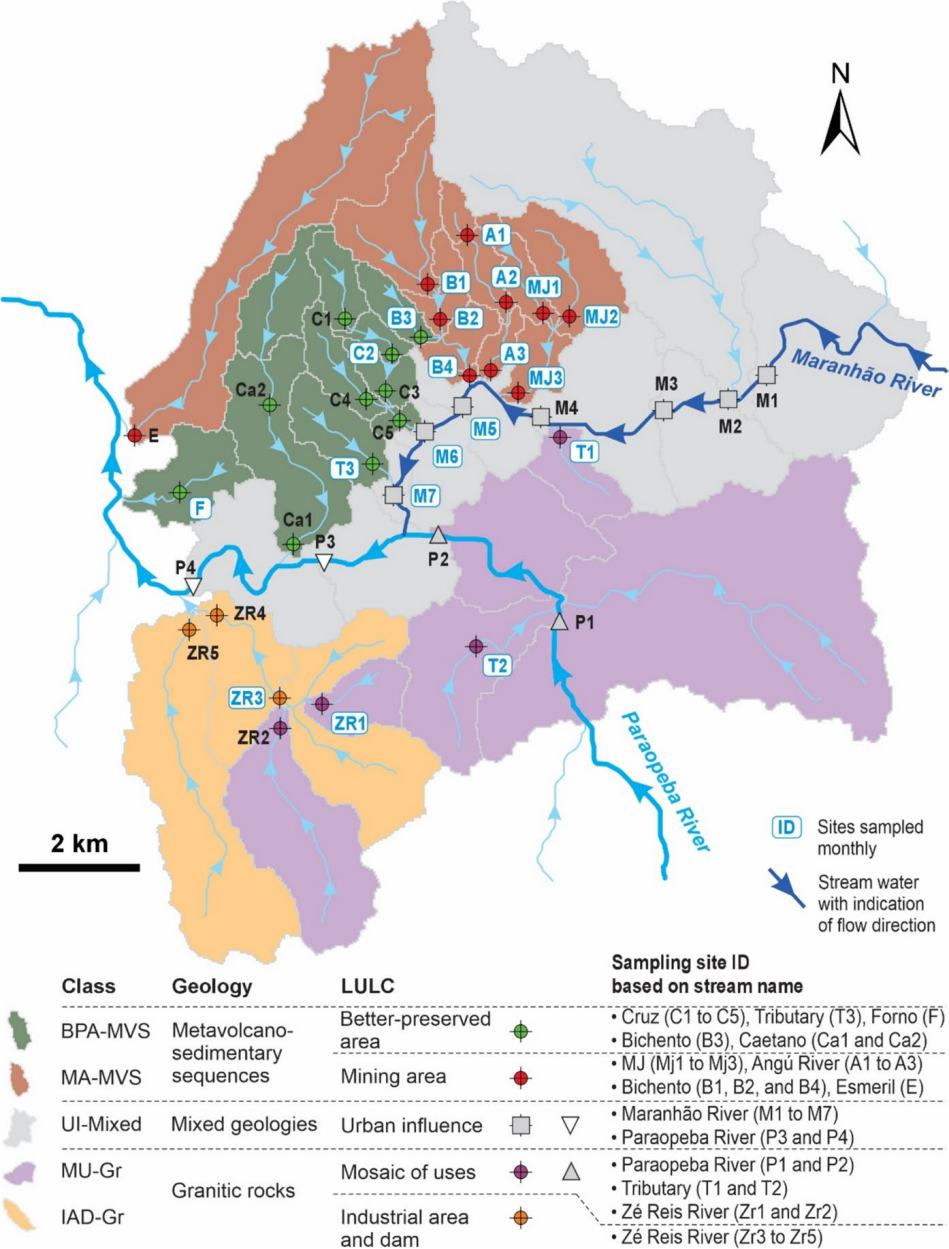
Schematic map of streamflow directions and surface water sampling site locations in the Congonhas Mineral District. Site IDs are based on stream names, and arrows indicate the direction of flow. The legend table exemplifies the catchment classification. Each sampling site was assigned to one of five geospatial classes based on the dominant geology and land use and land cover (LULC) within its upstream catchment area (see Fig. 1 for LULC and geological maps). Sites highlighted with a gray background were monitored monthly (January 2021–April 2024) as part of a long-term complementary dataset, while all sites were sampled quarterly for geochemical characterization (June 2022–April 2024)

A total of 38 surface water sampling points were considered and placed along the main watercourses of the CMD region (Fig. 2). The monitoring sites were selected based on catchment topography, the flow direction of main streams, geology, and LULC to assess the spatiotemporal changes in surface water quality related to human activities. Field campaigns were conducted monthly from January 2021 to April 2024, generally at regular intervals during the first two weeks of each month to ensure temporal consistency. Samples were classified according to the seasonality of the region (rainy season from November to April, and dry season from May to October).

Two sampling datasets were considered in this study regarding their main application:

- Main dataset for geochemical characterization: this dataset was designed to provide a comprehensive spatial characterization of surface water quality across the CMD and included all sampling points shown in Fig. 2. These sites were sampled and analyzed at three-month intervals between June 2022 and April 2024, totaling 8 field campaigns and covering two full hydrological cycles. The sampling strategy aimed to capture mainly spatial heterogeneity in water quality across different geological and LULC settings. Compared to the other data set, this dataset incorporated an expanded suite of analytical parameters (Table 1), including a wider range of physicochemical variables, major ions, and metals, thereby supporting hydrogeochemical characterization and comparative assessments.
- Complementary dataset for environmental monitoring: This is a long-term dataset originating from the operational monitoring program of the Viga Mine. It comprises monthly surface water quality data collected between January 2021 and April 2024 from 23 fixed monitoring stations located throughout the CMD (Fig. 2). This dataset emphasizes temporal resolution and consistency, making it particularly suited for detecting long-term trends and seasonal fluctuations. In contrast to the main dataset, the number of parameters analyzed was more restricted (Table 1), focusing on a core set of indicators essential for environmental monitoring.

**Table 1:** Water quality parameters, corresponding analytical methods, limits of quantification (LOQ), and units

Parameter	Analytical reference	Method/instrumentation	LOQ	Unit
Electrical conductivity (EC)^†^	APHA; 2510 B	In situ measurement with multiparameter probe	1.0	µS cm^−1^
Dissolved oxygen (DO)^†^	APHA; 4500-O G		0.1	mg L^−1^
pH^†^	APHA; 4500H + B		2 to 13	Not applicable
Turbidity (TB)^†^	APHA; 2130 B		0.1	NTU
Total alkalinity (Alk)^†^	APHA; 2320 B	Titration method	6.0	mg L^−1^
Total dissolved solids (TDS)^†^	APHA; 2540 C	TDS dried at 180ºC	5.0	mg L^−1^
Total suspended solids (TSS)^†^	APHA; 2540 D	TSS dried at 103–105 °C	5.0	mg L^−1^
Settleable solids (SS)^†^	APHA; 2540 F	Imhoff cone method	0.1	mL L^−1^
Total solids (TS)^†^	APHA; 2540 B and E	Gravimetric (TDS + TSS)	5.0	mg L^−1^
Thermotolerant coliforms (TC)	APHA; 9222 B and D	Membrane filtration and fecal coliform test	18	UFC 100 mL^−1^
Ca (Ca_T_/Ca_D_)*	USEPA; 6020 A/9811	Inductively coupled plasma atomic emission spectrometry (ICP-AES)	0.1	mg L^−1^
Mg (Mg_T_/Mg_D_)*	USEPA; 6020 A/9811		0.01	mg L^−1^
K (K_T_/K_D_)*	USEPA; 6020 A/9811		0.01	mg L^−1^
Na (Na_T_/Na_D_)*	USEPA; 6020 A/9811		0.1	mg L^−1^
Al (Al_T_/Al_D_)*	USEPA; 6020 A/9811		0.005	mg L^−1^
Fe (Fe_T_/Fe_D_)*^,†^	USEPA; 6020 A/9811		0.01	mg L^−1^
Mn (Mn_T_/Mn_D_)*^,†^	USEPA; 6020 A/9811		0.001	mg L^−1^
Total phosphorus (P)^†^	APHA; 4500 P, B, and E	Ascorbic acid method	0.0075	mg L^−1^
Chloride (Cl⁻)^†^	USEPA; 9056A 02/2007	Ion chromatography	0.5	mg L^−1^
Sulfate (SO_4_^2−^)	USEPA; 9056 A 02/2007		0.5	mg L^−1^
Nitrate (N–NO₃⁻)^†^	USEPA; 9056A 02/2007		0.11	mg L^−1^
Ammonia nitrogen (N-NH_3_^−^)^†^	APHA; 4500 NH_3_ F	Titrimetric method	0.1	mg L^−1^
Organic nitrogen (N_org_)^†^	APHA; 4500 N org B	digestion and distillation	0.5	mg L^−1^

‘^†^’ Parameters analyzed during the monthly campaigns; ‘*’ Parameters analyzed in both total and dissolved fractions; For metals, ‘T’ denotes to total concentration, and ‘D’ to dissolved concentration; All analytical methods follow standards established by the American Public Health Association (APHA, Awwa & WEF., 2017) and United States Environmental Protection Agency (United States Environmental Protection Agency (USEPA) 2007)

Sample collection and analytical procedures were conducted by a certified laboratory (ALS Ltda, Contagem-MG, Brazil), following internationally recognized quality assurance and quality control (QA/QC) protocols. Sample collection, preservation, and laboratory analyses were conducted in accordance with the American Public Health Association (APHA, Awwa & WEF., 2017) and United States Environmental Protection Agency (USEPA, 2007). A comprehensive list of measured water quality parameters, along with analytical methods, limits of quantification (LOQ), and measurement units, is provided in Table 1.

### Data processing, statistical analysis, and surface water characterization

The methodological procedures adopted in this study follow standard approaches that are widely applied in geochemical assessments (Calazans et al., 2018; Caritat & Cooper, 2016; Caritat et al., 2001; Chai et al., 2020; Reimann & Caritat, 2017; Reimann et al., 2018; Sahoo et al., 2019; Salomão et al., 2025). Initially, values below the LOQ were replaced with LOQ/2, while values above it were replaced with LOQ × 1.5. Each sampling site was classified into one of five geospatial classes based on the predominant LULC (MapBiomas, 2024) and geology (Silva et al., 2020) of its drainage area (see Figs. 1 and 2), field observations, and exploratory data analysis. These classes reflect contrasting environmental settings and anthropogenic pressures: (i) Better-preserved areas over metavolcano-sedimentary terrain (**BPA-MVS**): sites located in areas with predominantly natural vegetation, exhibiting limited anthropogenic influence, defined to reflect its role as a reference sector with a more conserved environmental setting within the CMD; (ii) Mining area over metavolcano-sedimentary terrain (**MA-MVS**): catchments directly influenced by mining infrastructure, such as open pits and waste deposits; (iii) Mixed use over granitic terrain (**MU-Gr**): areas with a predominance of pasturelands, interspersed with secondary vegetation and rural settlements; (iv) Industrial and dam area over granitic terrain (**IAD-Gr**): catchments influenced by industrial facilities and tailings dams; (v) Urban influence over mixed geology (**UI-Mixed**): drainage areas under the influence of urban centers, typically overlying complex lithological assemblages. The Mann–Whitney test was applied to assess seasonal differences in the dataset for each water quality parameter, and the Shapiro–Wilk test was applied to test data distribution. Descriptive statistics for each geospatial class are presented in Appendix A.

Hydrochemical characterization was carried out using Piper (1944), Gaillardet et al. (1999), and Gibbs (1970) diagrams to classify water types, identify dominant hydrogeochemical facies, and infer the main processes controlling water chemistry. These graphical methods enabled the assessment of the relative impacts of rock–water interactions, atmospheric inputs, and human activities on the observed chemical composition. Molar ratios were eventually expressed as median ± median absolute deviation.

Prior to multivariate analysis, missing values were replaced by the median for each parameter at each sampling site, preserving the proportional structure of the dataset. Linear Discriminant Analysis (LDA) was then applied to evaluate the extent to which the geospatial classification of sampling sites could be distinguished based on their hydrochemical signatures. This approach allowed for the identification of the most discriminant variables contributing to class separation and the assessment of classification accuracy. All statistical procedures were performed in the R programming environment (R Core Team, 2020) within RStudio (RStudio Team, 2020), using a combination of dedicated packages (Lorenz & Diekoff, 2017; Wickham, 2016).

### Determination of geochemical background and baseline values

Background and baseline values were determined using widely recognized methods described in the literature (Ander et al., 2013; Nakić et al., 2007; Reimann et al., 2018; Reimann & Caritat, 2005, 2017; Sahoo et al., 2019, 2020b; Salomão et al., 2018; Teixeira et al., 2020; USEPA, 2022a, 2022b), including Upper Tolerance Limits (UTL), Median + 2 × Median Absolute Deviation (mMAD), and Tukey Inner Fence (TIF). These values were calculated using custom scripts in the open-source software RStudio (RStudio Team, 2020), except for the UTL method, which was implemented using the software ProUCL (USEPA, 2022a, 2022b). The exploratory analyses supported the identification of distinct population types within the dataset, enabling the definition of reference values for specific areas, considering their relative land uses, in order to propose possible and realistic guideline values for the study area.

### Construction of spatial distribution maps for water quality indicators

Spatial distribution maps of key water quality indicators were created to assess seasonal patterns and spatial variability in the study area. Median values from a historical series covering two hydrological cycles were used to represent wet and dry seasons. Class intervals were defined through an exploratory hydrogeochemical mapping approach based on quantile or natural breaks methods, with spatial representation based on micro-watersheds or catchment areas (Dominech et al., 2025; Lancianese & Dinelli, 2015; Nezhad et al., 2017). All geographic datasets were standardized to the SIRGAS2000 geodetic reference system and processed in a Geographic Information System (GIS) environment using ArcGIS 10.8 (ESRI, 2016) and Quantum GIS (QGIS Development Team, 2009).

### Integration of UAV imagery and field data for environmental interpretation

High-resolution remote sensing imagery was captured using an uncrewed aerial vehicle (UAV) to supplement the spatial interpretation. UAV Imagery was collected with a DJI Mavic 2 Enterprise Advanced (DJI, 2025), equipped with an RGB camera and a radiometric thermal sensor. Flights were conducted over selected sampling sites with contrasting LULC and geology, following pre-planned flight routes. The RGB imagery was processed with photogrammetric techniques using Agisoft Metashape Professional. The UAV data helped refine LULC classification, enabling the identification of anthropogenic influences and the validation of geospatial patterns observed in water quality maps.

## Results and discussions

### Role of geology and LULC on surface water hydrogeochemical characteristics

Understanding the hydrogeochemical characteristics of surface water is crucial for protecting and managing watersheds. Here, we present the surface water hydrogeochemical characterization (Gaillardet et al., 1999; Gibbs, 1970; Piper, 1944) and general statistical tools for the main data set of the CMD region covering two hydrological cycles.

**Fig. 3:**
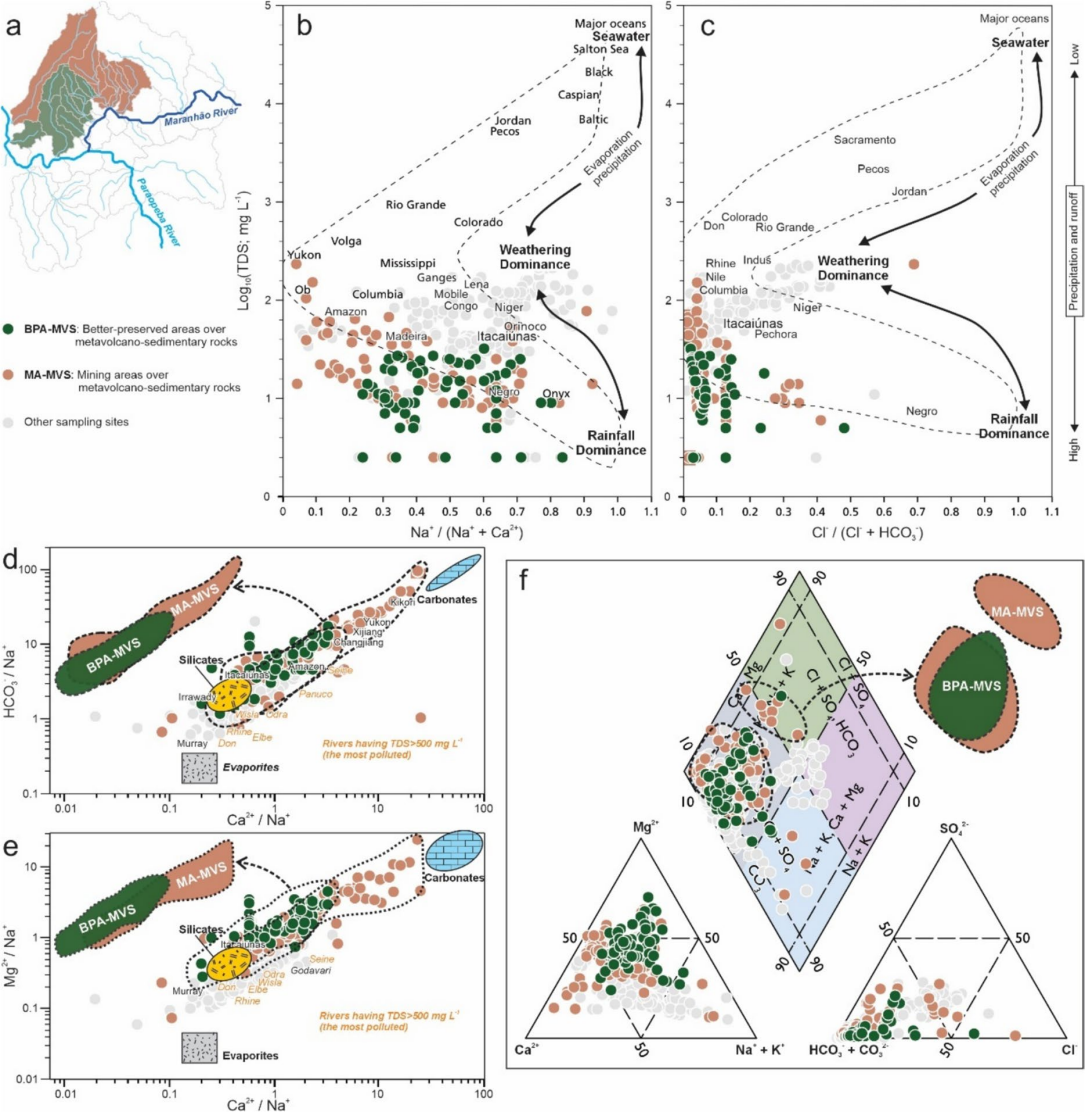
Hydrogeochemical characteristics of the surface water in the Congonhas Mineral District from **a** metavolcano-sedimentary terrain to the north, considering areas with better preserved vegetation cover (BPA-MVS; see Fig. 2) and mining (MA-MVS). **b**, **c** Gibbs diagram (molar ratios vs. total dissolved solids); **d**, **e** Gaillardet diagrams (Na-normalized molar ratios in the dissolved phase). Important rivers in the world are also plotted as references; **f** Piper diagram showing the main hydrochemical facies. Source: **b**, **c** based on Andrews et al. (2009) and Berner and Berner (1996) after Gibbs (1970); **d**, **e** modified from Gaillardet et al. (1999); **f** modified from Back and Hanshaw (1965) after Piper (1944)

**Fig. 4:**
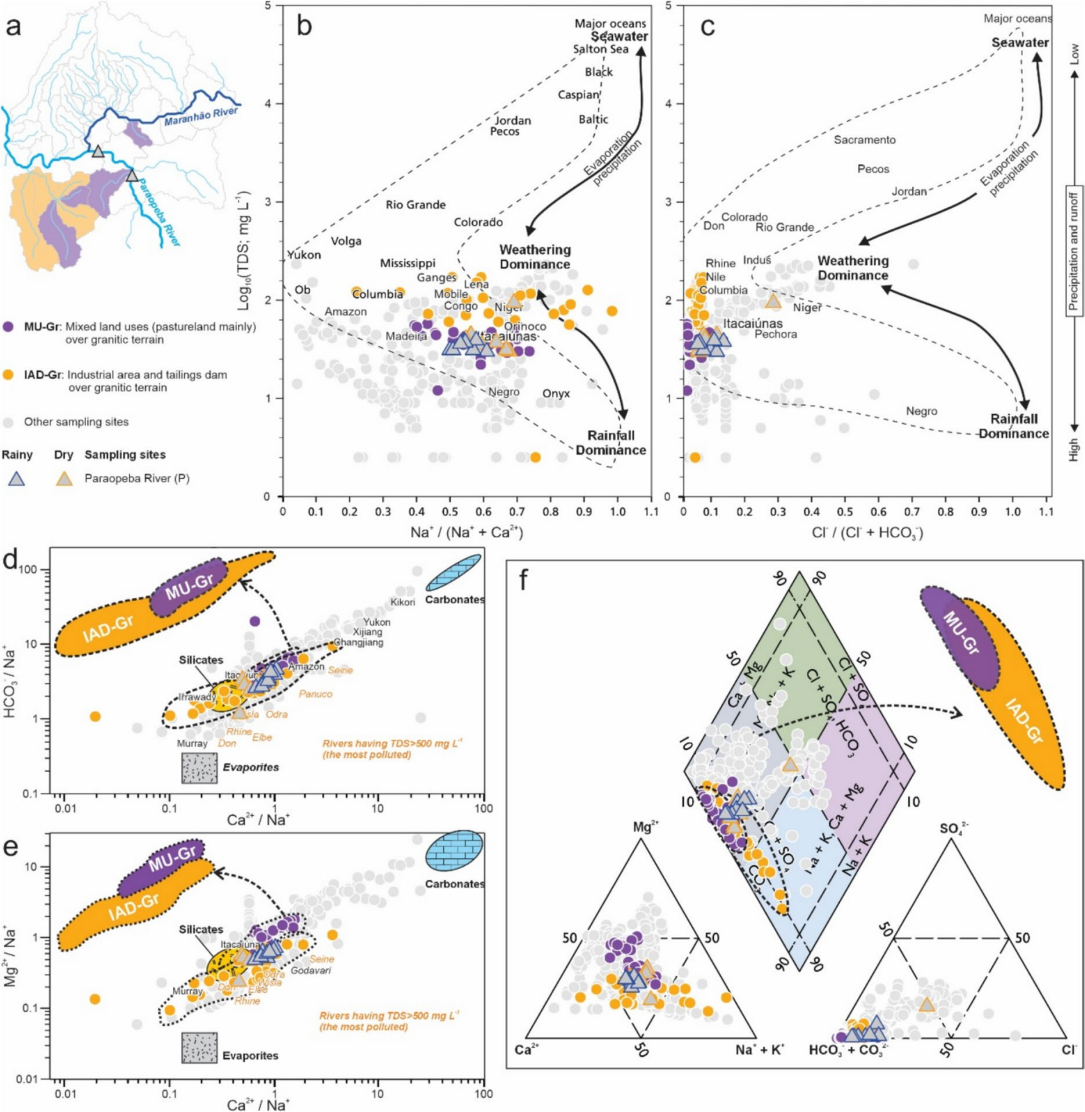
Hydrogeochemical characteristics of the surface water in the Congonhas Mineral District from **a** areas with mixed land uses, particularly dominated by pastureland (MU-Gr; see Fig. 2) and industrial areas and tailings dam (IAD-Gr) over granitic terrains in the south. **b**, **c** Gibbs diagram (molar ratios vs. total dissolved solids); **d**, **e** Gaillardet diagrams (Na-normalized molar ratios in the dissolved phase). Important rivers in the world are also plotted as references; **f** Piper diagram showing the main hydrochemical facies. Source: **b**, **c** based on Andrews et al. (2009) and Berner and Berner (1996) after Gibbs (1970); **d**, **e** modified from Gaillardet et al. (1999); **f** modified from Back and Hanshaw (1965) after Piper (1944)

**Fig. 5:**
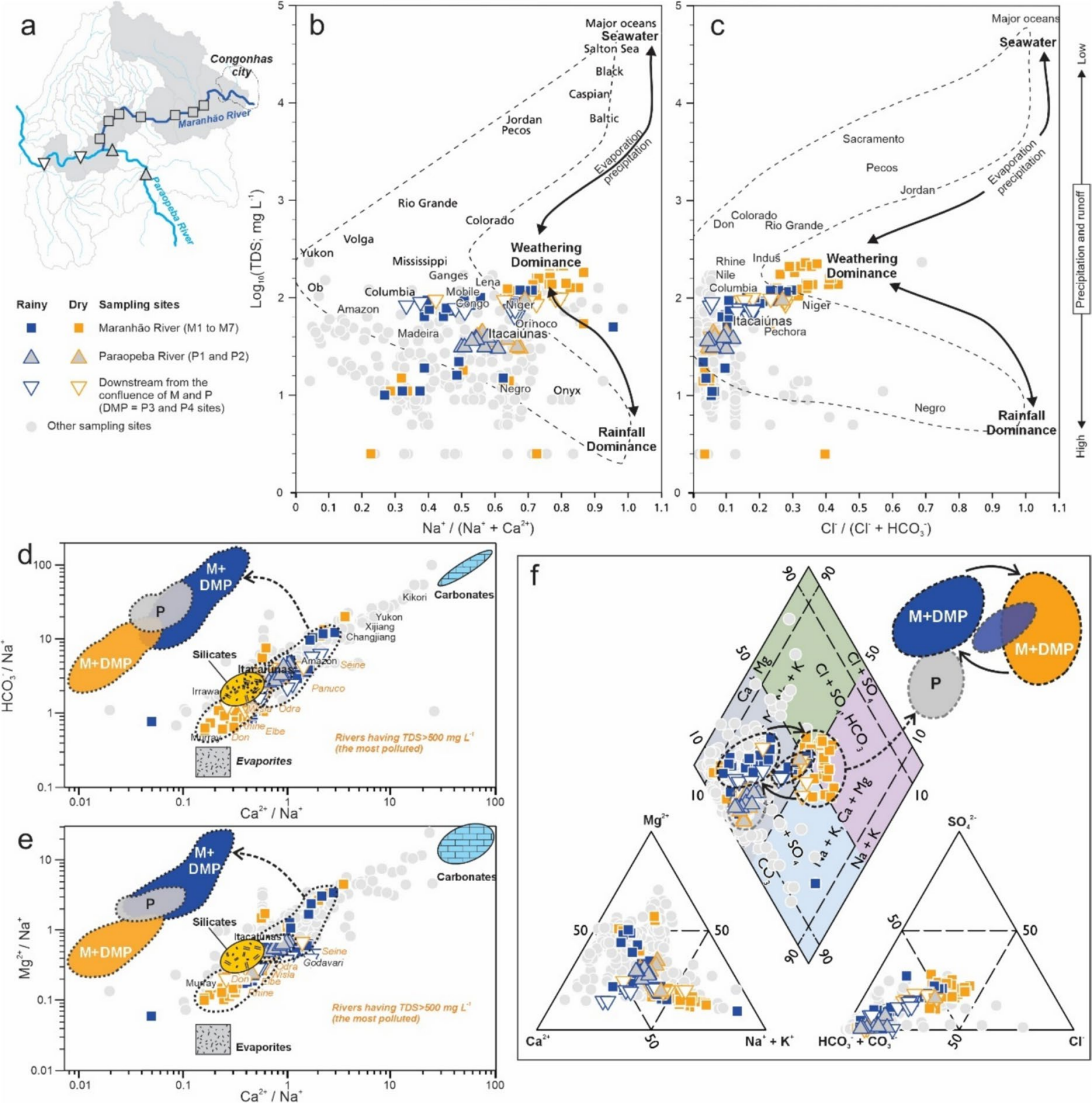
Hydrogeochemical characteristics of the surface water of the Congonhas Mineral District influenced by **a** urban areas over mixed geological contributions (UI-Mixed; see Fig. 2), along the main rivers (Paraopeba and Maranhão); Two sampling sites from mixed land uses, particularly dominated by pastureland (MU-Gr) are plotted for downstream reference of the Paraopeba River. **b**, **c** Gibbs diagram (molar ratios vs. total dissolved solids); **d**, **e** Gaillardet diagrams (Na-normalized molar ratios in the dissolved phase). Important rivers in the world are also plotted as references; **f** Piper diagram showing the main hydrochemical facies. Source: **b**, **c** based on Andrews et al. (2009) and Berner and Berner (1996) after Gibbs (1970); **d**, **e** modified from Gaillardet et al. (1999); **f** modified from Back and Hanshaw (1965) after Piper (1944)

The Gibbs diagram relates the TDS to the molar ratios Na^+^/(Na^+^ + Ca^2+^) and Cl^−^/(Cl^−^ + HCO_3_^−^), allowing the identification of the main environmental processes controlling the chemical composition of river waters, which are: (i) rainfall; (ii) rock weathering; and (iii) evaporation and/or crystallization. In general, the surface water samples from the study area plot predominantly between the weathering and precipitation domains (Figs. 3, 4, and 5). The Gaillardet diagram was applied to assess the lithological contribution (silicates, carbonates, and evaporates) to water chemistry, based on normalized cation concentrations (HCO_3_^−^/Na^+^ and Mg^2+^/Na^+^ vs. Ca^2+^/Na^+^). The results revealed that the majority of samples fell between the silicate and carbonate domains, with only a few plotting within the evaporate field (Figs. 3, 4, and 5). The Piper diagram demonstrates that the water samples of the study area are predominantly of the mixed bicarbonate type, with a small proportion showing alkali (Na^+^ and K^+^) and chloride (Cl^−^) enrichment (Figs. 3, 4, and 5).

Overall, the interpretations from the Gibbs, Gaillardet, and Piper diagrams indicate the important roles of lithology, rainfall, and land use in shaping the region's hydrogeochemistry. These findings are consistent with previous regional studies across the entire QF region (Almeida et al., 2025) and with local assessments (Paula et al., 2018). However, significant differences were observed when further evaluating and comparing the geospatial classes of the study area.

The five distinguished geospatial classes (Figs. 1 and 2), based on geological setting (Silva et al., 2020) and LULC (MapBiomas, 2024), revealed clear contrasts in hydrogeochemical characteristics and processes across the CMD region. Overall, consistent hydrogeochemical trends within these classes were observed, some convergent and others markedly distinct, reflecting the combined influence of geology and LULC. A small minority of samples fell outside the dominant trands, which is expected in a hydrochemically complex environment due to natural variability and local-scale processes. To explore these patterns, we present an integrated discussion according to the geospatial setting of the study area: BPA-MVS and MA-MVS classes in the northern sector (Fig. 3a); MU-Gr and IAD-Gr classes in the southern sector (Fig. 4a); and UI-Mixed areas along the Paraopeba and Maranhão rivers (Fig. 5a).

Regarding the Gibbs diagrams, the BPA-MVS and MA-MVS classes (Fig. 3b, c) are characterized, respectively, by low TDS (10 ± 7.4 and 15.5 ± 9.6), highly varying Na^+^/(Na^+^ + Ca^2+^) (0.49 ± 0.22 and 0.37 ± 0.27), and predominantly low to moderate Cl^−^/(Cl^−^ + HCO_3_^−^) (0.06 ± 0.05 and 0.04 ± 0.03). In contrast, the MU-Gr and IAD-Gr classes (Fig. 4b, c) are characterized, respectively, by moderate to high TDS (39 ± 10.4 and 84.5 ± 36.3), high Na^+^/(Na^+^ + Ca^2+^) (0.59 ± 0.12 and 0.61 ± 0.18), and low Cl^−^/(Cl^−^ + HCO_3_^−^) (0.01 ± 0.01 and 0.04 ± 0.01). This distribution reflects the combined effects on the hydrogeochemical composition of surface waters of geology, which is the major controlling factor, and seasonal rainfall patterns (Almeida et al., 2025).

Furthermore, the Gaillardet diagram offers valuable insights into the lithological controls on water chemistry. In the CMD region, the BPA-MVS and MA-MVS classes, located in the northern metavolcano-sedimentary terrain, display elevated Ca^2+^/Na^+^ (1.04 ± 0.72 and 1.69 ± 1.65) and Mg^2+^/Na^+^ (1.58 ± 0.76 and 1.60 ± 1.24) ratios, indicating a pronounced contribution from silicates and carbonate-rich rocks, the later probably containing minerals such as calcite, dolomite, and magnesite (Fig. 3d, e). These waters also exhibit high HCO₃⁻/Na⁺ ratios (4.54 ± 2.26 and 4.52 ± 4.16), suggesting intense rock-water interaction and dissolution processes involving CO_2_ from soils and the atmosphere (Costa et al., 2016; Salomão et al., 2023). These patterns are consistent with the presence of carbonatic and talc-enriched ultramafic rocks in the region (Oliveira et al., 2015; Parra et al., 2007; Roeser et al., 1980), as well as with Piper diagram results that classify these waters as mixed bicarbonate types (Fig. 3f).

In contrast, the southern classes, MU-Gr and IAD-Gr, located over granitic terrain, show lower Ca^2+^/Na^+^ (0.71 ± 0.31 and 0.63 ± 0.52) and Mg^2+^/Na^+^ ratios (0.90 ± 0.58 and 0.29 ± 0.19), with moderately lower HCO₃⁻/Na⁺ (3.77 ± 1.86 and 2.31 ± 1.07), reflecting reduced availability of divalent cations from silicate weathering (Fig. 4d, e). The more static behavior of MU-Gr samples, which are less influenced by the carbonates compared to the BPA-MVS and MA-MVS classes, and trend toward the silicate domain in the Gaillardet diagram, also reflects the low vegetation cover and limited input of organic acids due to the predominance of pastureland (Chai et al., 2020). Meanwhile, the IAD-Gr class displays broader geochemical variability, with several samples extending into the evaporite domain. According to the Piper diagram (Fig. 4f), samples from both MU-Gr and IAD-Gr are predominantly of the mixed bicarbonate type, with a strong trend toward alkali enrichment (Na^+^ and K^+^). This pattern is likely influenced by the high abundance of feldspars in the granitic bedrock, which release Na and K upon weathering (Adabanija et al., 2020; Nesbitt et al., 1996). However, the potential contribution of anthropogenic sources, such as industrial discharges and effluents from tailings dams, cannot be ignored, as these inputs are also known to elevate concentrations of these cations in surface waters (Candeias et al., 2015; Huang et al., 2016).

These observations highlight a clear geochemical contrast between the surface water of northern and southern sectors of the study area, driven primarily by the dominant local geology, metavolcano-sedimentary rocks in the north and granitic terrains in the south (Appendix B). LULC further amplifies these differences, with mining and better natural preserved areas in the north and anthropogenic pressures such as industry and pasture in the south modulating the geochemical pathways (Machado et al., 2024). Despite these contrasts, there is a notable overlap in the geochemical signatures of samples from different classes, which reflects the complex interplay between natural and anthropogenic drivers. These patterns are consistent with regional findings that underscore the dominant role of bedrock composition and land use in surface water composition (Almeida et al., 2025; Paula et al., 2018; Zhou et al., 2021).

A more complex behavior is observed in the UI-Mixed class, which encompasses the main fluvial systems of the region, including the Maranhão River (M1 to M7; see Fig. 2) and part of the Paraopeba River (P3 and P4; see Fig. 2) (Fig. 5a). Results from P1 and P2 sites were only included in hydrogeochemical diagrams for reference (Fig. 5a). Seasonally, the Maranhão River exhibits elevated TDS values, especially during the dry season (83 ± 26.7 mg/L and 126 ± 77.1 mg/L in in the rainy and dry season, respectively), along with high Na^+^/(Na^+^ + Ca^2+^) ratios (0.51 ± 0.18 and 0.74 ± 0.06) and Cl^−^/(Cl^−^ + HCO_3_^−^) ratios (0.14 ± 0.09 and 0.31 ± 0.08) (Fig. 5b, c). In contrast, the Paraopeba River upstream from the confluence maintains lower and more stable TDS levels (33.5 ± 4.5 mg/L in the rainy season and 38.5 ± 8.2 mg/L in the dry season), with moderately high Na^+^/(Na^+^ + Ca^2+^) ratios (0.54 ± 0.04 and 0.61 ± 0.08), and Cl^−^/(Cl^−^ + HCO_3_^−^) ratios of 0.09 ± 0.03 and 0.06 ± 0.02 (Fig. 5b, c). Downstream from the confluence, the water composition reflects the mixing of both rivers, with a notable increase in salinity and ionic contributions. TDS values reach 75.5 ± 8.15 mg/L in the rainy season and 93.5 ± 8.15 mg/L in the dry season, with corresponding increases in Na^+^/(Na^+^ + Ca^2+^) ratios (0.58 ± 0.13 and 0.66 ± 0.16) and Cl^−^/(Cl^−^ + HCO_3_^−^) ratios (0.15 ± 0.03 and 0.17 ± 0.13) (Fig. 5b, c).

In the Gibbs diagram (Fig. 5b, c), a seasonal trend is clearly observed. During the rainy season, most samples plot between the precipitation and rock weathering domains, whereas during the dry season, the Maranhão River samples distinctly shift toward the evaporation and/or crystallization domain (Gibbs, 1970). This behavior reflects a marked increase in salinity and suggests reduced dilution capacity combined with higher anthropogenic inputs during the dry period, typical of urban-impacted catchments (Costa et al., 2016; Jani & Toor, 2018; Osburn et al., 2016). Moreover, the Paraopeba River, downstream of its confluence with the Maranhão River, also displays increased TDS and a gradual shift toward the evaporation domain.

These geochemical trends are corroborated by the Gaillardet diagrams (Fig. 5d, e; Gaillardet et al., 1999), which show marked seasonal changes in ionic ratios. During the rainy season, Maranhão River samples exhibit moderate Ca^2^⁺/Na⁺ (0.97 ± 0.74), Mg^2^⁺/Na⁺ (0.48 ± 0.36), and HCO₃⁻/Na⁺ (2.66 ± 2.03) ratios, plotting in between the silicate and carbonate fields. However, in the dry season, these ratios drop substantially (Ca^2^⁺/Na⁺: 0.34 ± 0.10; Mg^2^⁺/Na⁺: 0.17 ± 0.07; HCO₃⁻/Na⁺: 0.90 ± 0.31) and move in the direction of the evaporite field, plotting more distant from the carbonates field. This indicates reduced rock-water interaction and enhanced ionic enrichment from non-geological sources. In contrast, the upper Paraopeba River exhibits more balanced ratios across seasons, consistent with a mixed carbonate–silicate signature typical of the region. After confluence, the downstream ratios reflect dilution and combination of both river waters, with intermediary values such as Ca^2^⁺/Na⁺: 0.76 ± 0.37 (rainy) and 0.51 ± 0.36 (dry), showing the transitional nature of these waters.

The Piper diagram (Fig. 5f) reveals that the Maranhão River shifts seasonally, presenting mixed bicarbonate-type water during the rainy season and trending toward Na and Cl-enriched mixed waters in the dry season. This seasonal shift is likely driven by increased contributions of domestic effluents (Calazans et al., 2018; Costa et al., 2016). Downstream from its confluence with the Paraopeba River, the mixed hydrochemical signature reflects both natural weathering and intensified anthropogenic influence.

These findings highlight the compositional heterogeneity within the UI-Mixed class and underscore the importance of both hydrological connectivity and human activity in changing surface water hydrogeochemistry. They also demonstrate that land use, seasonality, and urban proximity significantly modulate the ionic composition of river waters in the region (Silva et al., 2024a, 2024b; Sotiri et al., 2022; Wilbanks et al., 2023; Yevenes et al., 2018).

**Fig. 6:**
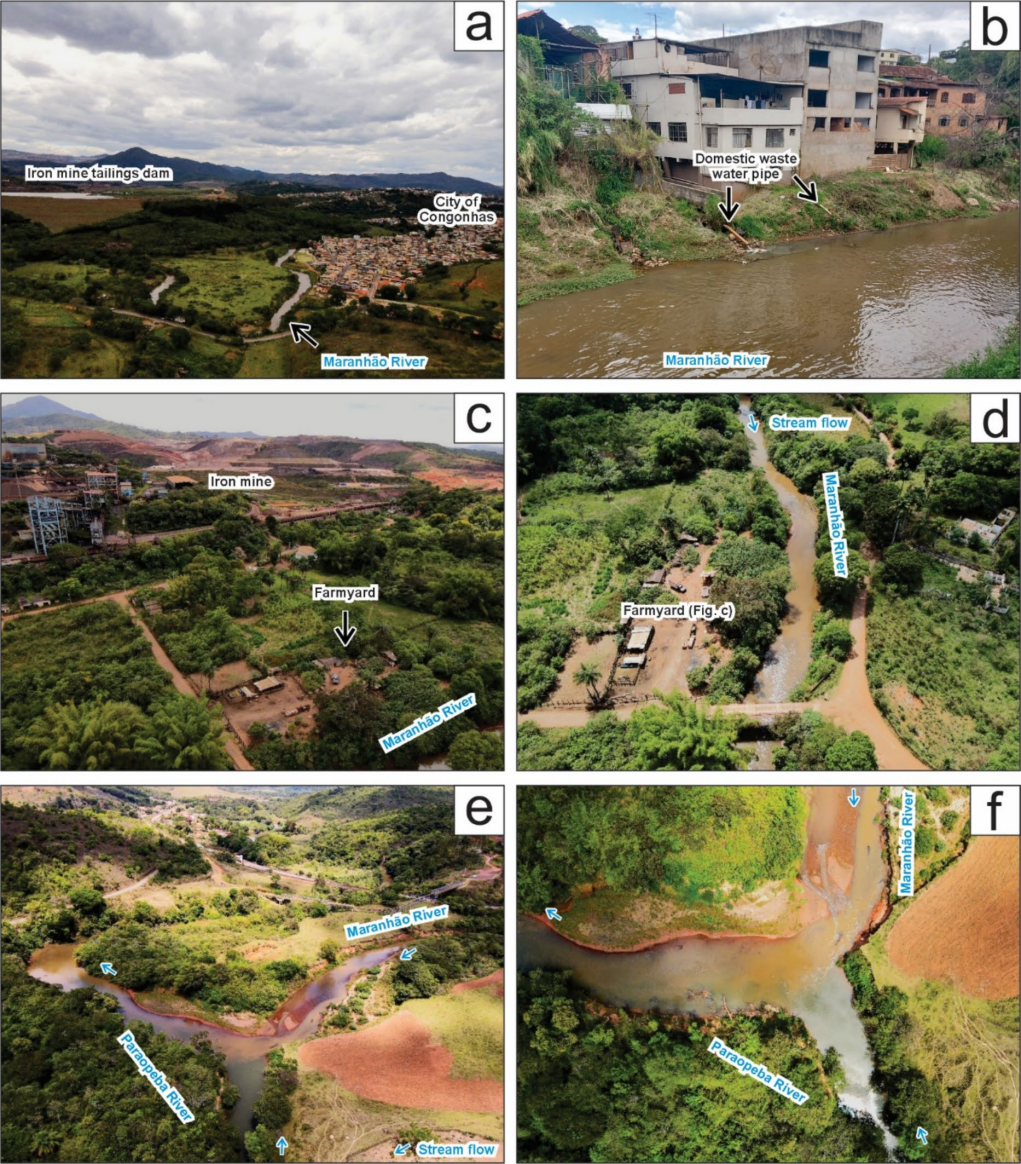
Anthropogenic and environmental aspects of the study area along the Maranhão River (MR). **a** Proximity of the MR to a tailing dam of an iron mine and the city of Congonhas; **b** Local scenario of urban settlement along MR in Congonhas, highlighting untreated domestic waste water been released to the river; **c**–**d** Iron mine in the background located in higher altitude areas and a farmyard with cattle corrals located near the floodplain of the MR; **e–f** The confluence of the Maranhão and Paraopeba rivers shows darker water with higher suspended sediments upon visual inspection in MR

In addition, field observations revealed that there is a clear anthropogenic contribution from the urban area to the water composition of rivers in the area (Fig. 6). Near the M1 sampling site (Fig. 2), it is possible to observe the proximity of the Maranhão River to a tailing dam and the urban area of Congonhas city (Fig. 6a). Further field observations revealed untreated domestic waste water been released to the Maranhão River (Fig. 6b), which is a common scene along the river within the urban area. In the intermediate part of the Maranhão River (between M3 and M4 sites, Fig. 2), the river flows through farmyards (Fig. 6c, d), where cattle corrals located at the floodplains represent potential sources of diffuse contamination of organic waste and sediments. Downstream, at the confluence of the Maranhão and Paraopeba rivers (between M7 and P2 sites; Fig. 2), a stark visual and olfactory contrast was documented (Fig. 6e, f). The Maranhão River appeared darker, with more suspended sediments upon visual inspection, and had a noticeable odor, while the Paraopeba River remained clearer, suggesting better water quality. (Fig. 6e, f).

**Fig. 7:**
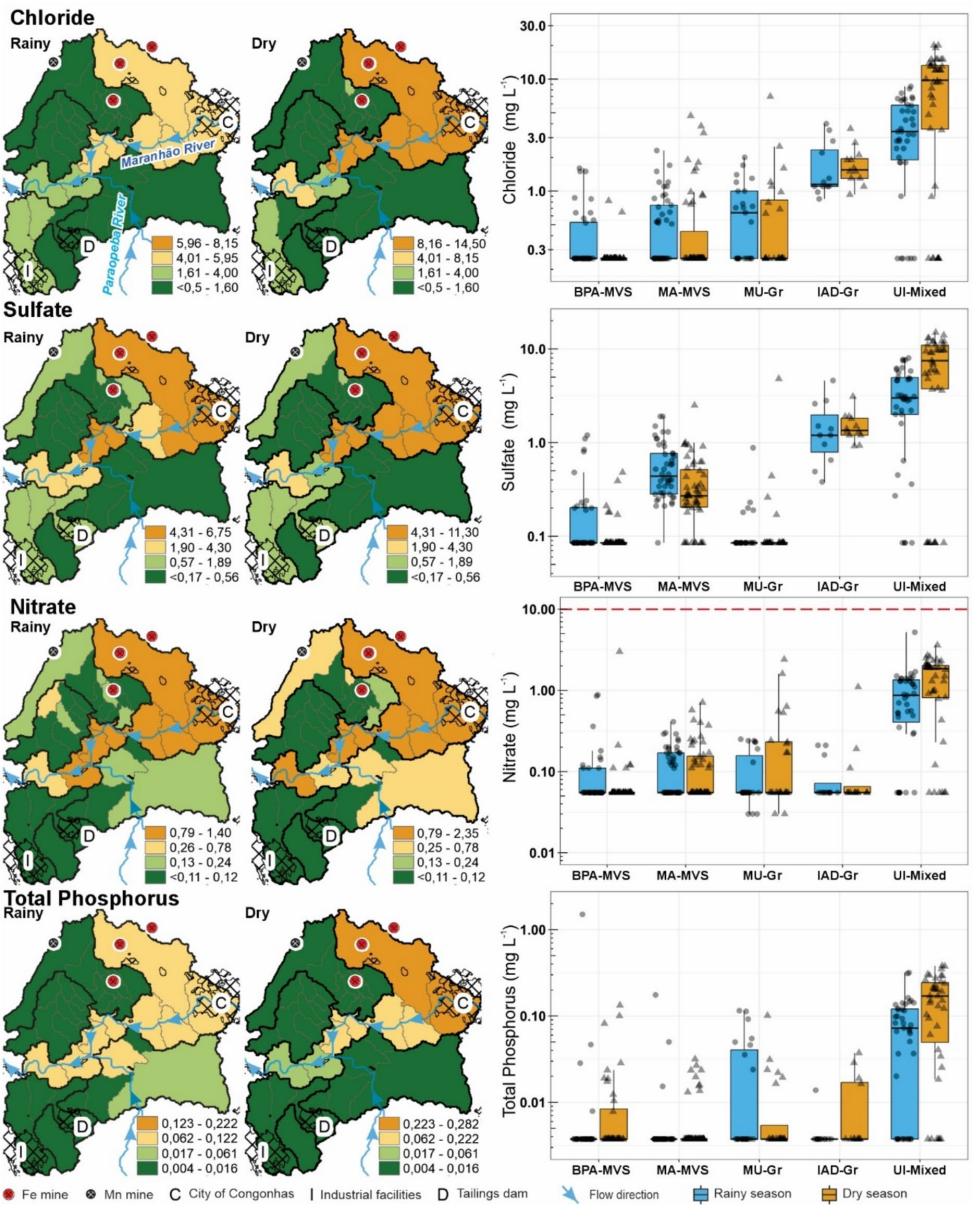
Spatial distribution maps of chloride, sulfate, nitrate, and total phosphorus, based on the median values from the entire historical series, during the rainy and dry seasons, in the study area. The boxplots represent all measurements from the sampling sites grouped according to their respective geospatial class (see Fig. 2): BPA-MVS: Better-preserved areas over metavolcano-sedimentary rocks; MA-MVS: Mining areas over metavolcano-sedimentary rocks; MU-Gr: Mixed land uses (pastureland) over granitic terrain; IAD-Gr: Industrial area and tailings over granitic terrain; UI-Mixed: Urban influence over mixed geology. The red dashed lines indicate the Class 2 freshwater regulatory limits for nitrate established by the Brazilian National Environment Council (CONAMA, 2005)

These observations are also supported by the spatial–temporal distribution maps of chloride, sulfate, nitrate, and phosphorus (Fig. 7), which are important parameters commonly associated with urban effluents (Silva et al., 2024a, 2024b; Sotiri et al., 2022; Tucci, 2008). These maps display a typical contaminant dissipation pattern, with higher concentrations immediately downstream of Congonhas, followed by gradual dilution along the flow path (Fig. 7). Other important water quality parameters also display a similar trend (TDS, TSS, SS, TS, TB, N_org_, and TC; see Appendix C). These trends are most evident during the dry season, when river flows are lower and pollutant concentrations are less diluted, except for TB, SS, TSS, and TS (see Appendix C).

Finally, the compiled evidence and interpretations strongly suggest that the Maranhão River is directly affected by urban effluents, which greatly change the chemical composition of the Paraopeba River downstream of their confluence. These rivers show different hydrogeochemical patterns, as demonstrated throughout this study. The environmental conditions observed in the Congonhas urban area provide clear evidence of human impact on water quality. Nevertheless, it is also important to consider the potential cumulative pressures from upstream sources in the upper Paraopeba River Basin (Fig. 1b), including effluents from other urban centers and land uses. This wider context may lead to higher contaminant loads, further reinforcing the need for integrated monitoring and management strategies at the watershed scale.

### Discriminant functions and geochemical drivers of class separation

**Fig. 8:**
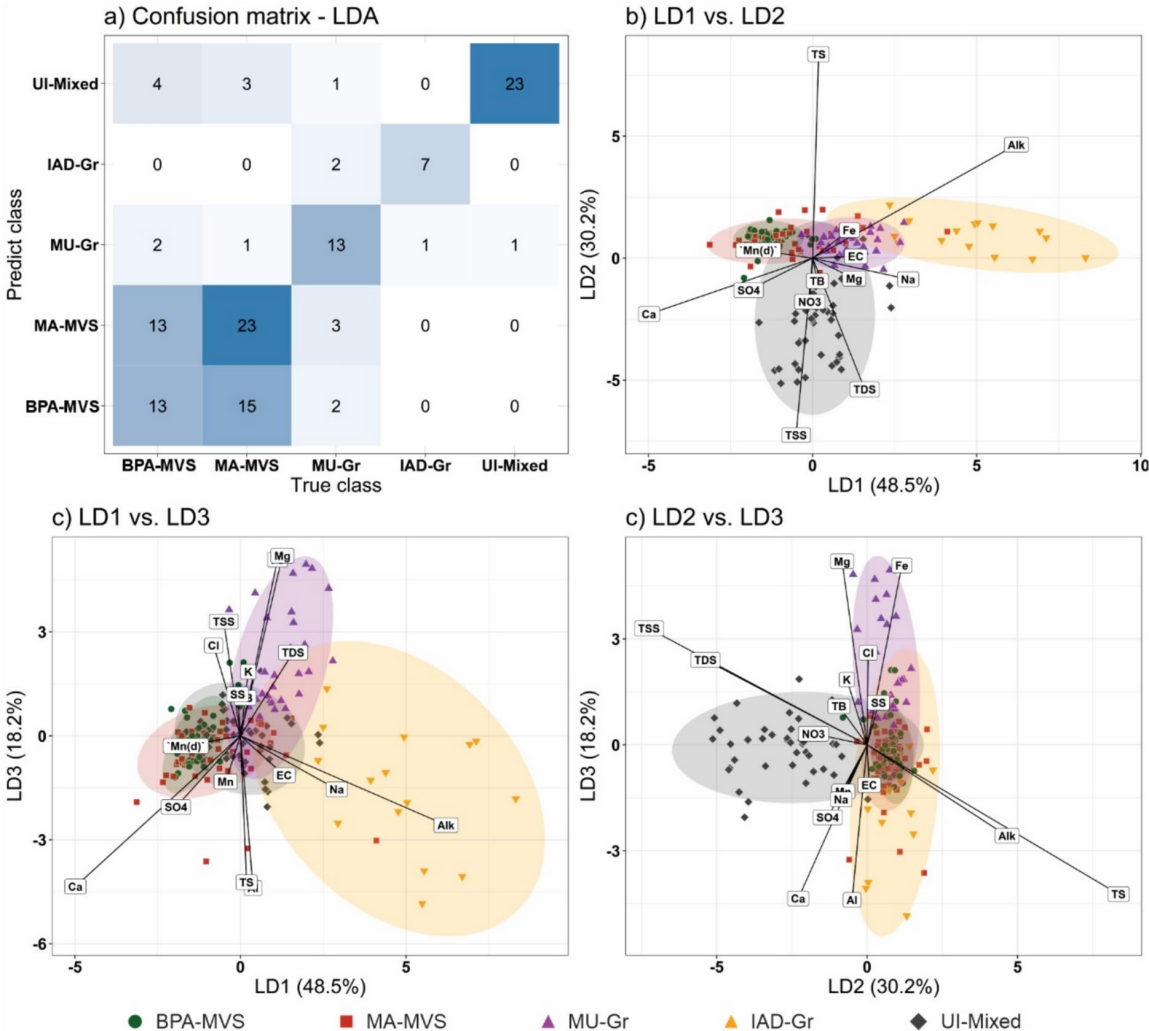
Results of the linear discriminant analysis (LDA) model for the five geospatial classes of the study area (Fig. 2). **a** Confusion matrix of the LDA. Scatter plots of the linear discriminant models: **b** LD1 vs. LD2; **c** LD1 vs. LD3; **d** LD2 vs. LD3; with their respective explained variances (%). Ellipses at a 0.05 significance level were included for each geospatial class for a better visualization. The second column presents the scaling vectors of the variables. Only variables with significant response (|x|> 0.3) are shown

To evaluate the LDA model's classification performance, a confusion matrix (Fig. 8a) was generated, and several performance metrics were computed. The overall accuracy was 62.2%, with a 95% confidence interval of 0.53–0.71 and a Kappa coefficient of 0.51, indicating moderate agreement. Despite being a linear model applied to a complex hydrogeochemical dataset, the LDA successfully discriminated most geospatial classes. Notably, MU-Gr, IAD-Gr, and UI-Mixed exhibited high sensitivity (0.72–0.78), moderate to excellent precision (0.62–0.96), and balanced accuracies above 0.82, reflecting well-defined hydrogeochemical signatures. The MA-MVS class also performed reasonably well, with balanced accuracy reaching 69%. However, BPA-MVS remained the most challenging to classify accurately, with the lowest sensitivity (0.43) and precision (0.41) among all classes. This highlights a consistent difficulty in distinguishing between BPA-MVS and MA-MVS (Fig. 8a), both of which are located over metavolcano-sedimentary terrains, a difficulty further explored in the following paragraph.

The coefficients of the linear discriminants (Fig. 8b, c, d) provide insight into the variables most effective for class separation. LD1 (48.5%) is mainly structured by strong positive loadings of Alk (2.07) and Na (0.97), with additional positive contributions from TDS (0.53), EC (0.44), Mg (0.42), and Fe (0.37). In contrast, Ca shows a strong negative loading (−1.66), followed by SO₄^2^⁻ (−0.65), dissolved Mn (−0.55). This indicates that the primary separation along LD1 reflects a gradient dominated by alkalinity and dissolved ion signatures vs. Ca-SO_4_^2−^. LD2 (30.2%) is dominated by TS (2.79) and Alk (1.55) on the positive side, while TSS (−2.42) and TDS (−1.79) contribute strongly on the negative side, with smaller contributions from Ca (−0.76) and NO_3_^−^ (−0.60), highlighting the relevance of suspended solids and nutrient loads in separating UI-Mixed and IAD-Gr from more preserved classes. LD3 (18.2%) shows strong positive contributions from Mg (1.73) and Fe (1.69), followed by TSS (1.10), Cl^−^ (0.87) and TDS (0.80), whereas Al (−1.46), Ca (−1.45), TS (−1.41) and Alk (−0.86) contribute negatively, reinforcing the role of lithological and contamination-related parameters. As LD4 accounted for only 3.0% of the total variance, it was considered negligible and thus excluded from further interpretation and visualization. These results indicate that a combination of lithology-dependent parameters (e.g., Alk, Ca, Fe, and Mg) and anthropogenic markers (e.g., Na, Cl⁻, SO₄^2^⁻, NH₃, TC) effectively discriminates surface water classes.

The BPA-MVS and MA-MVS classes exhibit consistently negative values for most parameters, notably Alk, Fe, Mn, Al, EC, and major cations, reflecting lower ionic strength and low anthropogenic input. The MA-MVS samples do not differ significantly from the BPA-MVS samples, due to the similar geology over rocks enriched in Mn oxides and sulfides (Appendix B). This occurs because both classes are genetically related by metavolcanosedimentary rocks, which contain phyllites, dolomitic layers, and BIFs with Mn concentrations in mineralized zones (Appendix B) (Dorr, 1969; Rosière & Chemale, 2000; Rossi, 2014).

In contrast, the UI-Mixed class exhibits positive scores for multiple constituents, particularly Na, Cl⁻, SO₄^2^⁻, P, N–NO₃⁻, N–NH₃⁻, and TC, reinforcing the pattern discussed in the previous section and corroborating the field observations (Appendix B). This combination of parameters reflects a strong urban influence, as wastewater discharges, surface runoff, and diffuse pollution sources typically enrich surface waters with nutrients, salts, and organic matter (Silva et al., 2024a, 2024b; Sotiri et al., 2022; Tucci, 2008).

The IAD-Gr class also displays elevated values of Alk, Na, TDS, and EC, likely reflecting lithological contributions from granitic terrain (Dorr, 1969; Rosière & Chemale, 2000), coupled with localized industrial pressures (Howladar et al., 2017; Singh et al., 2008). The MU-Gr class presents intermediate signatures with high Fe and Fe(d), moderate TB and EC, and negative values for Mn and nutrients, indicating a mixed lithological and land-use setting with limited urban or mining impact. Exposed soils accelerate erosive processes and natural geochemical influence is intensified in the cases of land uses such as pastures and mixed-use areas (Issaka & Ashraf, 2017; Li et al., 2024; Scalize et al., 2014).

The strong performance in identifying UI-Mixed, IAD-Gr, and MU-Gr classes reinforces the model’s sensitivity to urban, industrial, and geogenic signals. However, the overlap between BPA-MVS and MA-MVS persists, likely due to shared geological substrates and diffuse mining influence insufficiently captured by current predictors. Inclusion of trace elements and isotopic tracers may improve future discrimination.

### Environmental applications of background and baseline values in water management

Defining hydrogeochemical background and baseline values is a critical step in supporting water management in mining areas. These values are important to distinguish between naturally elevated concentrations and those influenced by anthropogenic pressures (Escher et al., 2015; Hao et al., 2024; Leão et al., 2019; Mulholland et al., 2012; Reimann & Garrett, 2005). Whereas the geochemical background reflects the admitted minimally disturbed environments, the geochemical baseline incorporates both natural and diffuse anthropogenic inputs, representing the overall condition (Albanese et al., 2007; Hinsby et al., 2008; Salminen & Gregorauskien, 2000; Shand et al., 2007). The exploratory analyses applied herein (see Topics 4.1 and 4.2) was essential to understand spatial and seasonal patterns, identify key geochemical indicators, and support the definition of reliable and spatially representative reference values. Furthermore, ongoing hydrogeochemical monitoring of the area, including LULC evaluation, and assessing the geological representativeness of each sampling site (Figs. 1 and 2), are essential for accurately selecting a group of samples to establish background and baseline values.

**Fig. 9:**
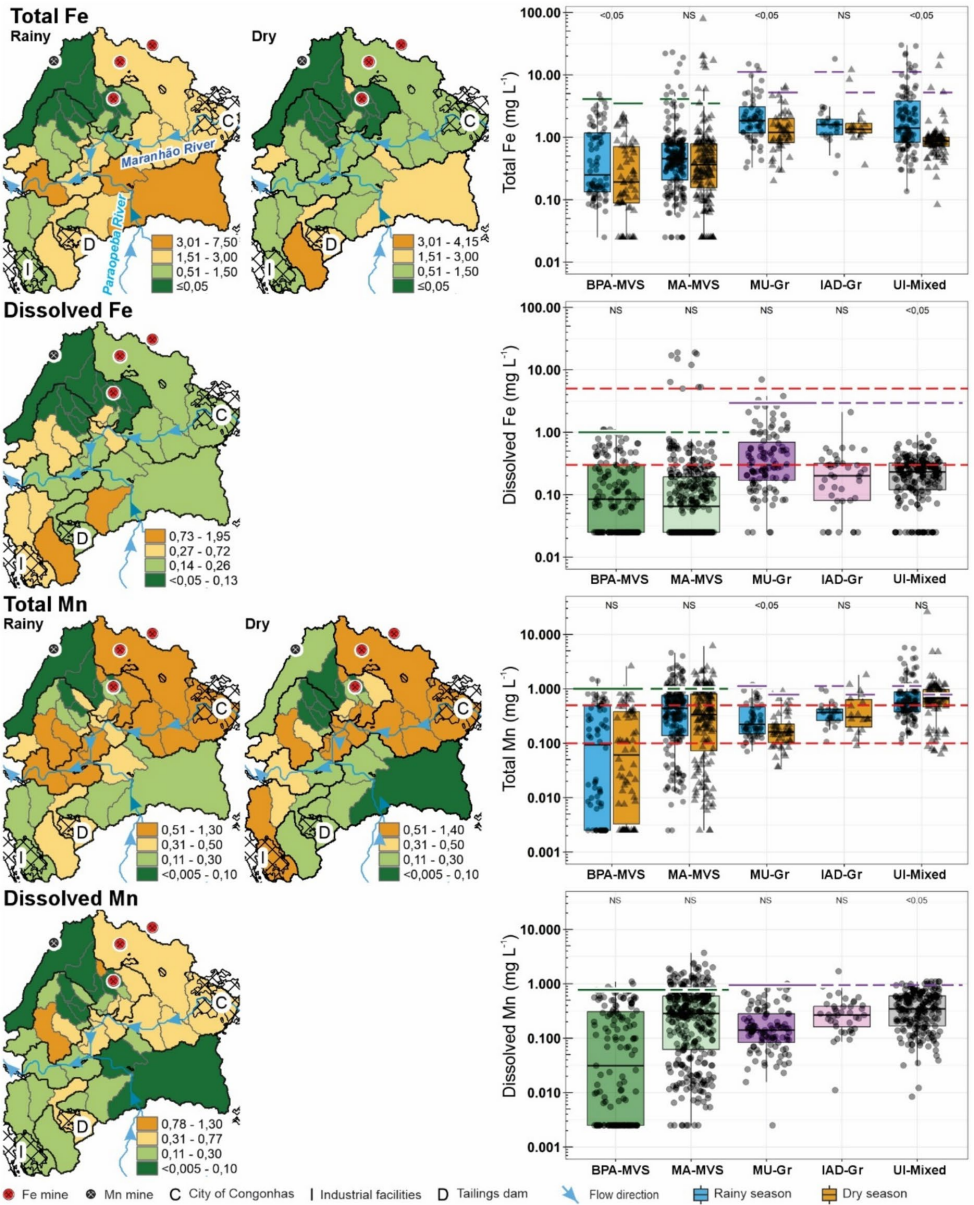
Spatial distribution maps of total and dissolved Fe and Mn, based on the median values from the entire historical series, during the rainy and dry seasons only for the total concentration, in the study area. The boxplots represent all measurements from the sampling sites grouped according to their respective geospatial class (see Fig. 2): BPA-MVS: Better-preserved areas over metavolcano-sedimentary rocks; MA-MVS: Mining areas over metavolcano-sedimentary rocks; MU-Gr: Mixed land uses (pastureland) over granitic terrain; IAD-Gr: Industrial area and tailings over granitic terrain; UI-Mixed: Urban influence over mixed geology. The *p*-value of the Mann–Whitney Test is displayed above the boxplots for each class to assess the statistical difference between the rainy and dry seasons (See Appendix A), if no differences were observer the NS (not significant) label is displayed. Solid lines indicate background values derived from BPA–MVS (green) and baseline values derived from MU–Gr (violet); dashed lines show the application of these reference values to MA–MVS (green) and to IAD–Gr and UI–Mixed (violet) (see Table 2). The red dashed lines indicate the Class 2 and 3 freshwater regulatory limits for dissolved Fe (0.3 and 5 mg L^−1^, respectively) and total Mn (0.1 and 0.5 mg L^−1^, respectively) established by the Brazilian National Environment Council (CONAMA, 2005)

In this study, the establishment of reference values for Fe and Mn estimated by a variety of methods (UTL, mMAD, and TIF; Table 2) and the construction of spatial distribution maps (Fig. 9) aimed to improve the geochemical characterization and enhance environmental interpretation in surface waters. Background values were derived from samples classified as BPA-MVS, representing minimally disturbed environments over metavolcano-sedimentary rocks in the northern portion of the study area, while baseline values were defined based on the MU-Gr class, which characterizes mixed land use over granitic terrain in the southern portion. Exclusively for the baseline calculation of the MU-Gr, data from sampling sites P1 and P2 were excluded as the results presented herein suggest significant natural and anthropogenic influences from upstream. These elements were selected because they are directly associated with Fe ore deposits and mining activities in the region, making them critical indicators for evaluating potential impacts on water quality in the CMD.

**Table 2:** Reference background and baseline concentrations of Fe and Mn in surface waters from the Congonhas Mineral District (CMD). Threshold values were calculated using the Upper Tolerance Limit (UTL), Median + 2*Median Absolute Deviation (mMAD), and Tukey Inner Fence (TIF) methods. When statistically significant seasonal variation was detected, values were calculated separately for the rainy (RS) and dry (DS) seasons, and are presented as paired values in the format (RS; DS). When no significant difference was observed, a single value is reported. For comparison, the table also includes background and baseline values from previous studies conducted in the Quadrilátero Ferrífero (QF) and in the Carajás Mineral Province (CMP), as well as environmental quality standards adopted by various national regulatory agencies

Region	Reference value	Method	Fe		Mn	
			Total	Dissolved	Total	Dissolved
Metavolcano-sedimentary terrain of the CMD—BPA-MVS samples (This study)	Background	UTL	(4.1; 3.49^b^)	1.00	1.00	0.77
		mMAD	(4.27; 3.80)	3.13	> max	> max
		TIF	(> max; > max)	12.71	> max	> max
Granitic terrain of the CMD—MU-Gr samples (This study)	Baseline	UTL	(11.18^b^; 5.22^c^)	2.95^b^	(1.13; 0.78^b^)	0.95^b^
		mMAD	(8.19; 4.91)	2.33	(1.02; 0.59)	0.99
		TIF	(13.00; 7.57)	5.74	(2.57; 0.72)	1.75
Quadrilátero Ferrífero (Almeida et al., 2025)	Baseline	mMAD	(7.28; 4.27)	(2.97; 2.35)	(0.78; 0.67)	(0.47; 0.76)
		TIF	(13.18; 7.23)	(6.59; 4.54)	(1.6; 1.28)	(0.77; 1.42)
Itacaiúnas River Watershed in (CMP) (Sahoo et al., 2019)	Baseline	mMAD	(7.76; 7.59)	–	(0.91; 1.55)	–
		TIF	(11.61; 13.96)	–	(1.91; 4.95)	–
Vicinity of N3 plateau—Fe deposit in CMP (Teixeira et al., 2020)	Background	UTL	0.92c	0.32	0.79	0.79
		mMAD	0.22	< 0.1	< 0.025	< 0.025
Vicinity of N4WSul plateau—Fe mine in CMP (Teixeira et al., 2020)	Background	UTL	1.3	0.45	1.02	0.84
		mMAD	1.14	0.62	0.13	0.05
Gelado Creek Watershed—Natural area in CMP (Salomão et al., 2025)	Background	UTL	(2.99; 1.24)	(0.73; 0.43)	(0.19; 0.08)	(0.14; 0.04)
		mMAD	(3.09; 0.88)	(0.6; 0.44)	(0.23; 0.08)	(0.23; 0.08)
Brazil (CONAMA, 2005)	Class 2	–	–	0.3	0.1	–
	Class 3	–	–	5	0.5	–
Canada (Canadian Council of Ministers of the Environment (CCME) 2019)	Short-term	–	–	–	–	3.60
	Long-term	–	–	–	–	0.43
Australia and New Zealand (Australian & New Zealand guidelines for fresh & marine water quality. Volume 1: The guidelines., 2000)	Freshwater guideline	–	–	0.3	–	1.90

Concentrations are expressed in mg L⁻^1^. ‘–’ not mentioned. ‘ > max’ results incompatible with the dataset, greater than the maximum obtained value. UTL results were calculated using the '95% BCA Bootstrap UTL with 95% coverage' method, assuming a non-parametric distribution, except for ‘b’, which used the '95% UTL with 95% coverage' method assuming a lognormal distribution, and ‘c’, which used the '95% HW UTL with 95% coverage' method assuming a Gamma distribution

Table 2 also presents a comparison of reference levels for Fe and Mn (in both total and dissolved forms) proposed for the CMD region with threshold concentrations obtained for the entire QF (Almeida et al., 2025), for different study cases in the Carajás Mineral Province (Sahoo et al., 2019; Salomão et al., 2025; Teixeira et al., 2020), which has important Fe deposits and world-class open-pit mines, and environmental quality standards for freshwater adopted by Brazil (CONAMA, 2005), Canada (Canadian Council of Ministers of the Environment (CCME) 2019), Australia, and New Zealand (Australian & New Zealand guidelines for fresh & marine water quality. Volume 1: The guidelines., 2000).

Each method used to derive background and baseline values has advantages and disadvantages. In terms of methodological concepts, the mMAD method is often considered a robust approach to identifying outliers, as it is less sensitive to outliers (Matschullat et al., 2000; Reimann & Filzmoser 2000; Reimann et al., 2018). The TIF method depends only on the data distribution and allows the definition of background values even if no outliers are present in the data set (Reimann et al., 2018). The threshold value can be higher than the maximum value in the dataset (Salomão et al., 2020). The UTL method is probabilistic in nature and strongly depends on data distribution, whether it is normal, lognormal, gamma, or nonparametric (USEPA 2022a, 2022b).

When comparing the results obtained from these different statistical methods for the BPA-MVS class, the TIF and, in part, mMAD methods delivered threshold values greater than the maximum value obtained (Table 2), limiting their application for environmental purposes. These methods were generally used in large-scale geochemical surveys (cf. Reimann et al., 2018; Salomão et al., 2020), which have a different configuration than high-frequency environmental monitoring (cf. Salomão et al., 2025; Teixeira et al., 2020). Further investigations should be carried out in order to understand the result of overestimating background values, which may be linked to the presence of undetected outliers, data transformation, or statistical limitations of a given dataset.

Among the methods tested, the UTL method yielded the most consistent and environmentally realistic results for the CMD. For instance, background concentrations derived from BPA-MVS samples for Fe _T_ are 4.1 mg L^−1^ during the rainy season and 3.49 mg L^−1^ during the dry season; Fe _D_ is 1.0 mg L^−1^, Mn _T_ is 1.0 mg L^−1^, and Mn _D_ is 0.77, which means that the seasonal variation in the last three is not significant. Baseline values for the MU-Gr class for Fe _T_ are 11.18 mg L^−1^ during the rainy season and 5.22 mg L^−1^ during the dry season; Fe _D_ is 2.95 mg L^−1^, Mn _T_ are 1.13 mg L^−1^ during the rainy season and 0.78 mg L^−1^ during the dry season; and Mn _D_ is 0.95 mg L^−1^. These values were also used to identify samples with high concentrations in boxplots, and their respective spatial distribution (Fig. 9).

Spatial distribution of Fe_T_ and Mn_T_ exhibit significant variations across geospatial groups and seasonal periods, reflecting the combined influence of natural and anthropogenic influences on surface water quality (Fig. 9), with a trend toward higher concentrations during the rainy season. This pattern suggests increased mobilization of Fe as oxide and hydroxide particles due to surface runoff and sediment resuspension during rainfall (Leão et al., 2019; Oliveira et al., 2015). Spatial distribution of Fe_D_ and Mn_D_ exhibited patterns distinct from the total fractions (Fig. 9). Overall, Fe_D_ concentrations were considerably lower than Fe_T_, confirming that Fe in the region predominantly occurs in particulate form, indicating low solubility under natural conditions. In contrast, Mn_D_ represented a higher proportion relative to Mn_T_, suggesting greater mobility of this element in aqueous medium, mainly in the colloidal form (Jaïry et al., 1999; Morgan & Stumm, 1964). This behavior may also be associated with the presence of Fe^2+^, which facilitates the release of Mn from iron oxides under alkaline pH conditions (Frierdich & Catalano, 2012).

Moreover, the highest frequencies of samples exceeding the UTL were recorded in MA-MVS and UI-Mixed (Fig. 9), highlighting the role of anthropogenic activities in the release of Fe and Mn to streams. These results indicate that, even considering the enrichment due to local lithologies (cf. Dorr, 1969; Rosière & Chemale, 2000; Rossi, 2014), such as the occurrences of BIF and manganiferous formation (Appendix B), mining operations and urban areas (e.g., Congonhas city) can contribute to enhancing metal fluxes to aquatic environments, particularly during periods of high precipitation (Calazans et al., 2018; Costa et al., 2016; Tucci, 2008). An additional hypothesis is that these elevated concentrations may also reflect cumulative upstream influences from the upper Paraopeba River (Fig. 1b, c), resulting from the presence of mineral deposits and Mn mining operations, as well as inputs from a larger urban center such as Conselheiro Lafaiete, which could further contribute to diffuse and point-source pollution pressures in the CMD.

Finally, the reference values derived here converge with the broader goal of refining water quality guidelines by offering a locally adjusted basis for comparison. This study has shown that Fe and Mn naturally have background and baseline concentrations exceeding regulatory limits due to geological factors and LULC current conditions. It is unreasonable to use regulatory values lower than natural background levels across different regions and ecosystems. The specific feature of each area should be considered for a proper investigation. Although the study established geochemical background and baseline values considering the local aspects of the study area, multiple samples exceeded these locally derived thresholds, demonstrating the influence of anthropogenic contributions to water quality. These thresholds can serve as practical geochemical guidelines to support water quality assessments, guide risk evaluations, and refine decision-making processes in environmental monitoring across the CMD.

## Conclusions

The integrated assessment successfully evaluated the multiple factors influencing hydrogeochemical baselines and identified sources of contamination in the Congonhas Mineral District. The main conclusions derived from this study are:

- Hydrogeochemical Controls: The chemical composition of surface waters in the CMD is predominantly controlled by a complex interplay between lithology, atmospheric precipitation, and land use. Graphical analysis confirmed that rock weathering and rainfall dilution are the primary natural processes, with silicate and carbonate dissolution being the most significant lithological contributions. The resulting waters are predominantly of the mixed bicarbonate type, with subordinate enrichment in Na⁺ + K⁺ and Cl⁻, indicative of specific anthropogenic or lithological influences.
- Spatial and Anthropogenic Patterns: A clear geospatial dichotomy exists between the northern metavolcano-sedimentary terrains (characterized by lower TDS and carbonate influences) and the southern granitic terrains (exhibiting higher TDS and alkali enrichment). Land use and urban proximity were identified as critical modulators of ionic composition, with areas of mining (MA-MVS), industry (IAD-Gr), and urban influence (UI-Mixed) displaying distinct and amplified hydrogeochemical signatures.
- The application of LDA showed good performance in capturing contrasts related to land use and occupation, reflecting well-defined hydrogeochemical signatures associated with urban, industrial, and lithological influences. The most relevant variables for class separation included lithogenic constituents such as Alk, Ca, Mg, and Fe, as well as anthropogenic markers such as Na, Cl⁻, SO₄^2^⁻, nutrients, and total carbon, confirming that the interaction between geology and environmental pressures controls hydrogeochemical variability. However, there was an overlap between BPA-MVS and MA-MVS classes due to the lithological similarity of metavolcano-sedimentary terrains enriched in Mn and BIF, which limited the model’s discriminating capacity. In this context, the inclusion of trace elements and isotopic tracers is suggested in future analyses to improve the distinction of overlapping classes.
- Regulatory implications and localized thresholds: The establishment of local geochemical background and baseline values for Fe and Mn is essential for accurate environmental assessment in the CMD. This study demonstrates that natural concentrations in the region can exceed generic national and international regulatory guidelines. Therefore, the use of locally derived, geology-specific reference values is a more realistic and effective approach for water quality management. These values provide a scientifically defensible basis for identifying anthropogenic contamination, guiding monitoring efforts, and informing regulatory decisions in mining-impacted watersheds.

In summary, this research underscores the necessity of adopting a geochemically informed and regionally tailored framework for environmental monitoring and regulation in mineral-rich provinces like the QF. The methodologies and findings presented provide a robust model for distinguishing between natural and anthropogenic contributions to water quality, ultimately supporting more sustainable resource management and the protection of aquatic ecosystems.

## Supplementary Information

Below is the link to the electronic supplementary material.

Supplementary file1 (DOCX 3002 kb)

## Data Availability

Data will be made available upon request.
