# Supplementary material for: Geology and land use as key drivers for hydrogeochemistry in a mining district of the Quadrilátero Ferrífero, Brazil: implications for water management strategies
Source: Environ Geochem Health. 2026 Jan 29;48(3):122. doi: 10.1007/s10653-026-02989-0 (PMC12855282; doi:10.1007/s10653-026-02989-0)

**Appendices**

**Manuscript Title:** Geology and land use as key drivers for hydrogeochemistry in a mining district of the Quadrilátero Ferrífero, Brazil: implications for water management strategies

**Authors:** Gabriel Negreiros Salomão*, Normara Yane Mar da Costa, Gabriel Soares de Almeida, Rafael Tarantino Amarante, Roberto Dall’Agnol, Paulo Rógenes Monteiro Pontes, Prafulla Kumar Sahoo, Lucas Pereira Leão, Eduardo Duarte Marques, Emmanoel Vieira da Silva-Filho

***Corresponding author email:** Gabriel.salomao@itv.org

**List of Appendices**

[Appendix A – Descriptive statistics of water quality parameters grouped by geospatial classes 2](#_Toc213663171)

[Appendix B – Spatial variations of the Congonhas Mineral District 7](#_Toc213663172)

[Appendix C – Spatial distribution maps of water quality indicators 8](#_Toc213663173)

# Appendix A – Descriptive statistics of water quality parameters grouped by geospatial classes

The p-value of the Mann-Whitney Test (pMW) was used to assess the statistical difference between the rainy (RS) and dry (DS) seasons. If no differences were found, the entire dataset (ED) was then chosen for analysis; p-value of the Shapiro-Wilk (pSW) for normality test; number of samples (n); percentage of samples below the limit of quantification (%<LQ); arithmetic mean (µ); standard deviation (s); coefficient of variation (CV); median absolute deviation (MAD); minimum (Min); percentile values (P25, P50, P75, P90, P95, and P98); maximum (Max), skewness, and kurtosis. ‘^†^’ Parameters analyzed during the monthly campaigns; For metals, ‘T’ denotes to total concentration, and ‘D’ to dissolved concentration;

## Better-preserved areas over metavolcano-sedimentary terrain (BPA-MVS).

| **Parameter** | **pMW** | **Dataset** | **pSW** | **n** | **% <LQ** | **µ** | **s** | **CV** | **MAD** | **Min** | **P25** | **P50** | **P75** | **P90** | **P95** | **P98** | **Max** | **Skewness** | **Kurtosis** |
| --- | --- | --- | --- | --- | --- | --- | --- | --- | --- | --- | --- | --- | --- | --- | --- | --- | --- | --- | --- |
| **Electrical conductivity (EC)^†^** | 0.14 | ED | <0.05 | 101 | 0 | 26.4 | 21.2 | 0.80 | 17.94 | 4.1 | 11.4 | 22.1 | 36.0 | 45.6 | 59.1 | 93.8 | 130.0 | 2.3 | 10.5 |
| **Dissolved oxygen (DO)^†^** | 0.11 | ED | <0.05 | 141 | 0 | 7.29 | 5.11 | 0.70 | 0.67 | 4.70 | 6.42 | 6.90 | 7.32 | 7.87 | 8.10 | 8.21 | 66.90 | 11.3 | 132.8 |
| **pH^†^** | 0.19 | ED | <0.05 | 141 | 0 | 7.00 | 0.49 | 0.07 | 0.49 | 5.18 | 6.67 | 7.06 | 7.33 | 7.64 | 7.74 | 7.88 | 7.90 | -0.4 | 3.3 |
| **Turbidity (TB)^†^** | <0.05 | RS | <0.05 | 74 | 0 | 17.75 | 36.00 | 2.03 | 4.08 | 0.44 | 1.95 | 4.02 | 18.00 | 37.08 | 78.19 | 130.78 | 232.00 | 3.9 | 20.4 |
|  |  | DS | <0.05 | 67 | 0 | 4.47 | 4.69 | 1.05 | 1.60 | 0.68 | 1.99 | 2.78 | 4.78 | 9.40 | 13.09 | 20.27 | 26.10 | 2.7 | 11.4 |
| **Total alkalinity (Alk)^†^** | 0.49 | ED | <0.05 | 140 | 45.0 | 8.30 | 6.23 | 0.75 | 5.18 | <6 | <6 | 6.50 | 13.21 | 17.69 | 19.59 | 21.59 | 28.06 | 1.0 | 3.0 |
| **Total dissolved solids (TDS)^†^** | 0.93 | ED | <0.05 | 140 | 16.4 | 14.45 | 12.21 | 0.84 | 11.12 | <5 | 5.00 | 11.00 | 22.00 | 27.10 | 32.15 | 41.44 | 77.00 | 2.0 | 9.3 |
| **Total suspended solids (TSS)^†^** | <0.05 | RS | <0.05 | 74 | 47.3 | 25.97 | 51.01 | 1.96 | 5.19 | <5 | <5 | 6.00 | 18.75 | 76.90 | 158.65 | 177.86 | 277.00 | 3.0 | 12.1 |
|  |  | DS | <0.05 | 66 | 66.7 | 7.21 | 9.05 | 1.26 | 0 | <5 | <5 | <5 | 9.50 | 19.50 | 23.75 | 33.70 | 49.00 | 2.5 | 9.9 |
| **Settleable solids (SS)^†^** | 0.29 | ED | <0.05 | 141 | 95.0 | 0.12 | 0.76 | 6.12 | 0 | <0.1 | <0.1 | <0.1 | <0.1 | <0.1 | <0.1 | 0.12 | 9.00 | 11.5 | 134.5 |
| **Total solids (TS)^†^** | 0.11 | ED | <0.05 | 140 | 10.7 | 31.75 | 41.84 | 1.32 | 19.27 | <5 | 9.00 | 20.00 | 38.50 | 61.50 | 101.95 | 182.22 | 302.00 | 3.5 | 18.0 |
| **Thermotolerant coliforms (TC)** | <0.05 | RS | <0.05 | 33 | 0 | 13219 | 11191 | 1 | 11861 | 45 | 1400 | 16000 | >16000 | >16000 | >16000 | >16000 | >16000 | -0.1 | 1.1 |
|  |  | DS | <0.05 | 40 | 7.5 | 4113 | 8493 | 2 | 697 | <18 | 62 | 490 | 1100 | >16000 | >16000 | >16000 | >16000 | 1.9 | 4.8 |
| **Ca _T_** | 0.38 | ED | <0.05 | 75 | 9.3 | 1.384 | 0.790 | 0.57 | 0.66 | <0.5 | 0.859 | 1.200 | 1.850 | 2.460 | 2.800 | 3.256 | 3.800 | 0.8 | 3.3 |
| **Ca _D_** | 0.69 | ED | <0.05 | 76 | 25.0 | 1.079 | 0.740 | 0.69 | 0.63 | <0.5 | <0.5 | 0.975 | 1.400 | 2.200 | 2.450 | 2.900 | 3.100 | 0.9 | 3.1 |
| **Mg _T_** | 0.41 | ED | <0.05 | 76 | 1.3 | 0.932 | 0.490 | 0.53 | 0.51 | <0.05 | 0.542 | 0.826 | 1.300 | 1.700 | 1.800 | 1.900 | 2.000 | 0.5 | 2.3 |
| **Mg _D_** | 0.21 | ED | <0.05 | 76 | 0 | 0.864 | 0.460 | 0.53 | 0.48 | 0.197 | 0.474 | 0.777 | 1.200 | 1.600 | 1.700 | 1.750 | 2.000 | 0.6 | 2.3 |
| **K _T_** | 0.79 | ED | <0.05 | 76 | 1.3 | 0.592 | 0.360 | 0.61 | 0.33 | <0.05 | 0.308 | 0.505 | 0.799 | 1.100 | 1.225 | 1.550 | 1.700 | 1.0 | 3.6 |
| **K _D_** | 0.85 | ED | <0.05 | 76 | 1.3 | 0.471 | 0.310 | 0.66 | 0.25 | <0.05 | 0.256 | 0.448 | 0.576 | 0.886 | 1.200 | 1.250 | 1.600 | 1.3 | 5.0 |
| **Na _T_** | 0.75 | ED | <0.05 | 76 | 26.3 | 0.930 | 0.750 | 0.81 | 0.65 | <0.5 | <0.5 | 0.759 | 1.200 | 1.700 | 2.150 | 3.500 | 3.900 | 2.0 | 7.8 |
| **Na _D_** | 0.26 | ED | <0.05 | 76 | 54.0 | 0.595 | 0.490 | 0.82 | 0 | <0.5 | <0.5 | <0.5 | 0.770 | 1.300 | 1.725 | 1.950 | 2.300 | 1.6 | 5.0 |
| **Al _T_** | <0.05 | RS | <0.05 | 36 | 2.8 | 0.202 | 0.300 | 1.46 | 0.05 | <0.025 | 0.066 | 0.083 | 0.198 | 0.485 | 0.816 | 1.120 | 1.400 | 2.8 | 10.1 |
|  |  | DS | <0.05 | 40 | 37.5 | 0.050 | 0.050 | 0.96 | 0.05 | <0.025 | <0.025 | 0.045 | 0.070 | 0.094 | 0.102 | 0.181 | 0.264 | 2.5 | 11.4 |
| **Al _D_** | 0.27 | WD | <0.05 | 75 | 74.7 | <0.025 | 0.020 | 0.84 | 0 | <0.025 | <0.025 | <0.025 | <0.025 | 0.042 | 0.056 | 0.079 | 0.094 | 2.3 | 7.9 |
| **Fe _T_^†^** | <0.05 | RS | <0.05 | 74 | 1.4 | 0.852 | 1.080 | 1.27 | 0.25 | <0.05 | 0.134 | 0.253 | 1.175 | 2.370 | 3.070 | 3.870 | 4.900 | 1.7 | 5.6 |
|  |  | DS | <0.05 | 60 | 16.7 | 0.485 | 0.610 | 1.26 | 0.25 | <0.05 | 0.089 | 0.192 | 0.712 | 1.600 | 1.905 | 2.082 | 2.300 | 1.6 | 4.4 |
| **Fe _D_^†^** | 0.48 | ED | <0.05 | 140 | 37.1 | 0.212 | 0.260 | 1.23 | 0.09 | <0.05 | <0.05 | 0.085 | 0.302 | 0.607 | 0.786 | 1.022 | 1.100 | 1.7 | 5.2 |
| **Mn _T_^†^** | 0.87 | ED | <0.05 | 140 | 26.4 | 0.248 | 0.370 | 1.48 | 0.11 | <0.005 | <0.005 | 0.075 | 0.418 | 0.688 | 0.819 | 1.088 | 2.600 | 2.7 | 14.7 |
| **Mn _D_^†^** | 0.51 | ED | <0.05 | 141 | 35.5 | 0.187 | 0.250 | 1.36 | 0.04 | <0.005 | <0.005 | 0.031 | 0.307 | 0.568 | 0.730 | 0.799 | 1.100 | 1.4 | 4.1 |
| **Total phosphorus (P)^†^** | <0.05 | RS | <0.05 | 74 | 90.5 | 0.030 | 0.180 | 5.90 | 0 | <0.0075 | <0.0075 | <0.0075 | <0.0075 | <0.0075 | 0.054 | 0.153 | 1.500 | 8.2 | 68.9 |
|  |  | DS | <0.05 | 66 | 78.8 | 0.011 | 0.020 | 2.02 | 0 | <0.0075 | <0.0075 | <0.0075 | <0.0075 | 0.021 | 0.035 | 0.095 | 0.133 | 4.1 | 19.9 |
| **Chloride (Cl⁻)^†^** | <0.05 | RS | <0.05 | 74 | 75.7 | <0.5 | 0.32 | 0.79 | 0 | <0.5 | <0.5 | <0.5 | <0.5 | 0.78 | 1.05 | 1.50 | 1.60 | 2.3 | 7.9 |
|  |  | DS | <0.05 | 66 | 90.9 | <0.5 | 0.12 | 0.42 | 0 | <0.5 | <0.5 | <0.5 | <0.5 | <0.5 | 0.63 | 0.66 | 0.82 | 3.1 | 11.3 |
| **Sulfate (SO_4_^2-^)** | <0.05 | RS | <0.05 | 36 | 66.7 | 0.22 | 0.29 | 1.32 | 0 | <0.17 | <0.17 | <0.17 | 0.20 | 0.64 | 0.91 | 1.13 | 1.20 | 2.4 | 7.3 |
|  |  | DS | <0.05 | 40 | 85.0 | <0.17 | 0.08 | 0.73 | 0 | <0.17 | <0.17 | <0.17 | <0.17 | 0.18 | 0.22 | 0.41 | 0.48 | 3.4 | 14.4 |
| **Nitrate (N-NO₃⁻)^†^** | 0.8 | ED | <0.05 | 137 | 83.9 | <0.11 | 0.27 | 2.75 | 0 | <0.11 | <0.11 | <0.11 | <0.11 | 0.12 | 0.16 | 0.50 | 3.00 | 9.4 | 98.9 |
| **Ammonia nitrogen (N-NH_3_^-^)^†^** | <0.05 | RS | <0.05 | 74 | 83.8 | 0.06 | 0.12 | 2.01 | 0 | <0.05 | <0.05 | <0.05 | <0.05 | 0.13 | 0.21 | 0.41 | 0.86 | 5.0 | 30.4 |
|  |  | DS | <0.05 | 66 | 93.9 | <0.05 | 0.01 | 0.53 | 0 | <0.05 | <0.05 | <0.05 | <0.05 | <0.05 | <0.05 | 0.07 | 0.13 | 5.6 | 36.4 |
| **Organic nitrogen (N_org_)^†^** | 0.61 | ED | <0.05 | 140 | 83.6 | <0.5 | 0.94 | 2.06 | 0 | <0.5 | <0.5 | <0.5 | <0.5 | 0.67 | 1.25 | 2.89 | 10.00 | 8.1 | 78.0 |

## Mining area over metavolcano-sedimentary terrain (MA-MVS)

| **Parameter** | **pMW** | **Dataset** | **pSW** | **n** | **% <LQ** | **µ** | **s** | **CV** | **MAD** | **Min** | **P25** | **P50** | **P75** | **P90** | **P95** | **P98** | **Max** | **Skewness** | **Kurtosis** |
| --- | --- | --- | --- | --- | --- | --- | --- | --- | --- | --- | --- | --- | --- | --- | --- | --- | --- | --- | --- |
| **Electrical conductivity (EC)^†^** | 0.33 | ED | <0.05 | 185 | 0 | 57.8 | 71.1 | 1.2 | 29.2 | 7.4 | 17.8 | 34.0 | 71.0 | 111.6 | 200.7 | 330.8 | 427.0 | 3.0 | 13.0 |
| **Dissolved oxygen (DO)^†^** | 0.74 | ED | <0.05 | 327 | 0 | 6.66 | 0.75 | 0.11 | 0.73 | 2.90 | 6.20 | 6.70 | 7.14 | 7.56 | 7.80 | 8.00 | 8.57 | -1.1 | 7.5 |
| **pH^†^** | 0.24 | ED | <0.05 | 355 | 0 | 7.22 | 0.49 | 0.07 | 0.52 | 5.93 | 6.90 | 7.23 | 7.59 | 7.85 | 7.94 | 8.03 | 8.36 | -0.3 | 2.6 |
| **Turbidity (TB)^†^** | <0.05 | RS | <0.05 | 176 | 0 | 46.64 | 125.77 | 2.70 | 16.34 | 0.31 | 4.38 | 13.15 | 40.75 | 89.80 | 188.30 | 308.00 | 1390.00 | 7.8 | 77.5 |
|  |  | DS | <0.05 | 150 | 0 | 14.68 | 20.91 | 1.42 | 5.24 | 0.10 | 2.55 | 5.44 | 18.33 | 41.95 | 58.55 | 86.22 | 113.00 | 2.6 | 10.0 |
| **Total alkalinity (Alk)^†^** | <0.05 | RS | <0.05 | 177 | 32.8 | 18.94 | 23.63 | 1.25 | 13.20 | <6 | <6 | 11.90 | 27.00 | 36.13 | 54.23 | 73.03 | 177.33 | 3.7 | 22.7 |
|  |  | DS | <0.05 | 151 | 19.9 | 31.79 | 46.30 | 1.46 | 16.80 | <6 | 7.33 | 14.33 | 31.00 | 93.63 | 148.96 | 175.39 | 251.83 | 2.7 | 10.3 |
| **Total dissolved solids (TDS)^†^** | 0.75 | ED | <0.05 | 360 | 2.2 | 36.72 | 45.51 | 1.24 | 17.79 | <5 | 12.00 | 21.00 | 44.00 | 72.00 | 129.05 | 222.92 | 315.00 | 3.2 | 14.6 |
| **Total suspended solids (TSS)^†^** | <0.05 | RS | <0.05 | 176 | 38.6 | 36.14 | 95.48 | 2.64 | 10.38 | <5 | <5 | 9.50 | 27.00 | 72.00 | 126.00 | 405.00 | 840.00 | 5.7 | 39.6 |
|  |  | DS | <0.05 | 151 | 51.0 | 17.41 | 43.77 | 2.51 | 0 | <5 | <5 | <5 | 18.00 | 33.00 | 54.00 | 103.00 | 441.00 | 7.1 | 63.2 |
| **Settleable solids (SS)^†^** | 0.58 | ED | <0.05 | 327 | 85.9 | <0.1 | 0.26 | 3.03 | 0 | <0.1 | <0.1 | <0.1 | <0.1 | 0.10 | 0.20 | 0.50 | 4.50 | 14.9 | 246.8 |
| **Total solids (TS)^†^** | 0.22 | ED | <0.05 | 327 | 0.3 | 66.28 | 97.89 | 1.48 | 32.62 | <5 | 17.00 | 38.00 | 67.50 | 149.40 | 235.10 | 323.32 | 961.00 | 4.6 | 31.9 |
| **Thermotolerant coliforms (TC)** | <0.05 | RS | <0.05 | 44 | 6.8 | 11133 | 11352 | 1 | 4138 | <18 | 350 | 2800 | >16000 | >16000 | >16000 | >16000 | >16000 | 0.2 | 1.1 |
|  |  | DS | <0.05 | 51 | 11.8 | 5047 | 9475 | 2 | 297 | <18 | 45 | 220 | >16000 | >16000 | >16000 | >16000 | >16000 | 1.5 | 3.3 |
| **Ca _T_** | 0.85 | ED | <0.05 | 97 | 5.2 | 4.119 | 6.300 | 1.530 | 1.379 | <0.5 | 1.100 | 1.800 | 5.400 | 8.700 | 10.600 | 23.080 | 49.000 | 4.5 | 29.4 |
| **Ca _D_** | 0.56 | ED | <0.05 | 96 | 10.4 | 3.668 | 5.890 | 1.610 | 1.443 | <0.5 | 0.813 | 1.500 | 4.875 | 7.750 | 10.500 | 18.200 | 47.000 | 4.8 | 32.7 |
| **Mg _T_** | 0.32 | ED | <0.05 | 97 | 0 | 1.689 | 1.380 | 0.820 | 0.861 | 0.390 | 0.593 | 1.100 | 2.500 | 3.900 | 4.420 | 5.300 | 5.500 | 1.1 | 3.1 |
| **Mg _D_** | 0.41 | ED | <0.05 | 96 | 0 | 1.508 | 1.290 | 0.850 | 0.780 | 0.285 | 0.526 | 1.000 | 2.400 | 3.450 | 4.000 | 5.300 | 5.400 | 1.2 | 3.7 |
| **K _T_** | 0.20 | ED | <0.05 | 97 | 4.1 | 0.585 | 0.580 | 1.000 | 0.259 | <0.05 | 0.263 | 0.434 | 0.641 | 0.984 | 1.820 | 2.524 | 3.600 | 2.9 | 12.3 |
| **K _D_** | 0.13 | ED | <0.05 | 96 | 3.1 | 0.554 | 1.190 | 2.150 | 0.222 | <0.05 | 0.183 | 0.304 | 0.524 | 0.739 | 1.650 | 2.600 | 11.000 | 7.4 | 63.5 |
| **Na _T_** | 0.59 | ED | <0.05 | 96 | 17.7 | 1.325 | 1.990 | 1.500 | 0.492 | <0.5 | 0.661 | 0.976 | 1.400 | 1.850 | 2.875 | 5.710 | 18.000 | 6.6 | 53.4 |
| **Na _D_** | 0.26 | ED | <0.05 | 96 | 36.5 | 0.974 | 1.930 | 1.990 | 0.658 | <0.5 | <0.5 | 0.694 | 0.970 | 1.400 | 1.775 | 4.320 | 18.000 | 7.5 | 64.7 |
| **Al _T_** | <0.05 | RS | <0.05 | 46 | 10.9 | 0.105 | 0.160 | 1.500 | 0.032 | <0.025 | 0.050 | 0.065 | 0.089 | 0.140 | 0.424 | 0.573 | 0.915 | 3.9 | 18.1 |
|  |  | DS | <0.05 | 51 | 31.4 | 0.055 | 0.050 | 0.830 | 0.057 | <0.025 | <0.025 | 0.051 | 0.077 | 0.096 | 0.107 | 0.155 | 0.266 | 2.1 | 10.3 |
| **Al _D_** | 0.10 | WD | <0.05 | 96 | 78.1 | <0.025 | 0.020 | 0.820 | 0 | <0.025 | <0.025 | <0.025 | <0.025 | 0.040 | 0.066 | 0.074 | 0.080 | 2.2 | 6.8 |
| **Fe _T_^†^** | 0.08 | ED | <0.05 | 324 | 5.6 | 1.472 | 5.400 | 3.670 | 0.432 | <0.05 | 0.185 | 0.418 | 0.795 | 1.970 | 5.225 | 16.540 | 79.000 | 10.2 | 135.8 |
| **Fe _D_^†^** | 0.32 | ED | <0.05 | 329 | 47.1 | 0.498 | 2.320 | 4.650 | 0.059 | <0.05 | <0.05 | 0.065 | 0.193 | 0.473 | 0.642 | 5.784 | 19.000 | 6.8 | 49.7 |
| **Mn _T_^†^** | 0.21 | ED | <0.05 | 329 | 1.5 | 0.584 | 0.700 | 1.190 | 0.479 | <0.005 | 0.116 | 0.370 | 0.770 | 1.300 | 1.860 | 2.344 | 6.100 | 3.1 | 19.2 |
| **Mn _D_^†^** | 0.50 | ED | <0.05 | 328 | 2.4 | 0.429 | 0.490 | 1.150 | 0.372 | <0.005 | 0.062 | 0.286 | 0.597 | 1.100 | 1.365 | 1.900 | 3.700 | 2.2 | 10.6 |
| **Total phosphorus (P)^†^** | <0.05 | RS | <0.05 | 178 | 84.3 | 0.015 | 0.030 | 2.310 | 0 | <0.0075 | <0.0075 | <0.0075 | <0.0075 | 0.045 | 0.092 | 0.149 | 0.205 | 3.6 | 16.2 |
|  |  | DS | <0.05 | 151 | 84.8 | 0.014 | 0.040 | 2.770 | 0 | <0.0075 | <0.0075 | <0.0075 | <0.0075 | 0.024 | 0.057 | 0.152 | 0.299 | 5.1 | 30.7 |
| **Chloride (Cl⁻)^†^** | 0.63 | ED | <0.05 | 327 | 66.7 | 0.62 | 0.77 | 1.25 | 0 | <0.5 | <0.5 | <0.5 | 0.67 | 1.54 | 2.07 | 3.65 | 4.70 | 2.9 | 11.7 |
| **Sulfate (SO_4_^2-^)** | <0.05 | RS | <0.05 | 46 | 2.2 | 0.61 | 0.44 | 0.72 | 0.27 | <0.17 | 0.28 | 0.44 | 0.77 | 1.25 | 1.45 | 1.90 | 1.90 | 1.4 | 4.2 |
|  |  | DS | <0.05 | 51 | 17.7 | 0.41 | 0.40 | 0.98 | 0.13 | <0.17 | 0.21 | 0.27 | 0.52 | 0.91 | 0.96 | 1.00 | 2.50 | 3.1 | 16.3 |
| **Nitrate (N-NO₃⁻)^†^** | 0.11 | ED | <0.05 | 328 | 54.3 | 0.17 | 0.41 | 2.39 | 0.04 | <0.11 | <0.11 | <0.11 | 0.17 | 0.27 | 0.43 | 0.88 | 5.00 | 9.0 | 98.3 |
| **Ammonia nitrogen (N-NH_3_^-^)^†^** | 0.74 | ED | <0.05 | 327 | 80.4 | 0.10 | 0.38 | 3.66 | 0 | <0.05 | <0.05 | <0.05 | <0.05 | 0.17 | 0.41 | 0.77 | 5.29 | 10.0 | 123.1 |
| **Organic nitrogen (N_org_)^†^** | <0.05 | RS | <0.05 | 176 | 68.8 | <0.5 | 0.78 | 1.68 | 0 | <0.5 | <0.5 | <0.5 | 0.58 | 0.82 | 1.06 | 1.37 | 9.91 | 10.3 | 124.9 |
|  |  | DS | <0.05 | 151 | 60.3 | 0.74 | 1.21 | 1.63 | 0.07 | <0.5 | <0.5 | <0.5 | 0.67 | 1.43 | 2.45 | 5.47 | 8.24 | 3.9 | 19.6 |

## Mixed use over granitic terrain (MU-Gr)

| **Parameter** | **pMW** | **Dataset** | **pSW** | **n** | **% <LQ** | **µ** | **s** | **CV** | **MAD** | **Min** | **P25** | **P50** | **P75** | **P90** | **P95** | **P98** | **Max** | **Skewness** | **Kurtosis** |
| --- | --- | --- | --- | --- | --- | --- | --- | --- | --- | --- | --- | --- | --- | --- | --- | --- | --- | --- | --- |
| **Electrical conductivity (EC)^†^** | 0.44 | ED | <0.05 | 67 | 0 | 56.4 | 20.6 | 0.4 | 18.1 | 15.2 | 42.9 | 52.9 | 67.0 | 78.8 | 86.5 | 90.8 | 151.0 | 1.4 | 8.0 |
| **Dissolved oxygen (DO)^†^** | 0.10 | ED | <0.05 | 107 | 0 | 6.57 | 0.49 | 0.07 | 0.47 | 5.07 | 6.20 | 6.50 | 6.90 | 7.22 | 7.39 | 7.49 | 8.10 | 0.2 | 3.5 |
| **pH^†^** | 0.61 | ED | <0.05 | 107 | 0 | 7.10 | 0.43 | 0.06 | 0.43 | 6.00 | 6.85 | 7.19 | 7.39 | 7.62 | 7.74 | 7.86 | 7.90 | -0.5 | 3.0 |
| **Turbidity (TB)^†^** | <0.05 | RS | <0.05 | 56 | 0 | 44.53 | 94.81 | 2.13 | 17.07 | 2.10 | 9.05 | 17.35 | 33.30 | 72.45 | 156.03 | 404.80 | 518.00 | 3.9 | 17.5 |
|  |  | DS | <0.05 | 51 | 0 | 13.49 | 15.53 | 1.15 | 7.68 | 1.80 | 5.09 | 10.30 | 14.15 | 21.15 | 40.00 | 59.80 | 89.70 | 3.3 | 14.7 |
| **Total alkalinity (Alk)^†^** | 0.05 | ED | <0.05 | 107 | 0.9 | 30.24 | 8.63 | 0.29 | 7.41 | <6 | 24.42 | 28.33 | 36.00 | 42.60 | 46.33 | 47.46 | 51.83 | 0.2 | 3.3 |
| **Total dissolved solids (TDS)^†^** | 0.81 | ED | <0.05 | 107 | 0 | 39.16 | 12.38 | 0.32 | 10.38 | 12.00 | 31.00 | 38.00 | 44.00 | 54.40 | 57.70 | 68.16 | 94.00 | 1.1 | 6.0 |
| **Total suspended solids (TSS)^†^** | <0.05 | RS | <0.05 | 56 | 10.7 | 45.21 | 98.82 | 2.19 | 15.57 | <5 | 9.50 | 17.00 | 32.00 | 69.50 | 175.50 | 431.40 | 564.00 | 4.1 | 19.7 |
|  |  | DS | <0.05 | 51 | 25.5 | 15.01 | 14.46 | 0.96 | 13.34 | <5 | <5 | 12.00 | 20.50 | 28.00 | 38.00 | 55.00 | 76.00 | 2.0 | 8.2 |
| **Settleable solids (SS)^†^** | 0.60 | ED | <0.05 | 107 | 73.8 | 0.13 | 0.29 | 2.27 | 0 | <0.1 | <0.1 | <0.1 | 0.10 | 0.20 | 0.30 | 0.98 | 2.00 | 5.4 | 33.4 |
| **Total solids (TS)^†^** | 0.10 | ED | <0.05 | 107 | 0 | 70.90 | 73.23 | 1.03 | 17.79 | 24.00 | 45.50 | 56.00 | 70.50 | 91.00 | 122.70 | 274.12 | 596.00 | 5.4 | 35.5 |
| **Thermotolerant coliforms (TC)** | <0.05 | RS | <0.05 | 23 | 0 | 17468 | 10564 | 1 | 0 | 55 | 8550 | >16000 | >16000 | >16000 | >16000 | >16000 | >16000 | -1.0 | 2.1 |
|  |  | DS | <0.05 | 24 | 0 | 6690 | 10264 | 2 | 526 | 45 | 193 | 400 | 8625 | >16000 | >16000 | >16000 | >16000 | 1.1 | 2.3 |
| **Ca _T_** | 0.61 | ED | <0.05 | 47 | 0 | 4.770 | 1.650 | 0.350 | 1.186 | 2.600 | 3.700 | 4.500 | 5.450 | 6.800 | 7.500 | 8.516 | 11.000 | 1.5 | 5.9 |
| **Ca _D_** | 0.96 | ED | <0.05 | 47 | 0 | 4.234 | 1.400 | 0.330 | 1.038 | 2.400 | 3.400 | 3.700 | 4.950 | 6.040 | 7.240 | 7.596 | 8.700 | 1.3 | 4.3 |
| **Mg _T_** | 0.62 | ED | <0.05 | 47 | 0 | 2.957 | 1.180 | 0.400 | 0.890 | 1.500 | 2.050 | 2.400 | 3.850 | 4.540 | 5.300 | 5.624 | 5.900 | 0.9 | 2.7 |
| **Mg _D_** | 0.14 | ED | <0.05 | 47 | 0 | 2.617 | 1.100 | 0.420 | 0.741 | 1.300 | 1.800 | 2.100 | 3.450 | 4.180 | 4.570 | 5.200 | 5.200 | 0.9 | 2.5 |
| **K _T_** | <0.05 | RS | 0.31 | 23 | 4.4 | 1.411 | 0.700 | 0.490 | 0.363 | <0.05 | 1.000 | 1.200 | 2.100 | 2.280 | 2.390 | 2.624 | 2.800 | 0.2 | 2.4 |
|  |  | DS | <0.05 | 24 | 0 | 1.083 | 0.620 | 0.570 | 0.302 | 0.169 | 0.823 | 0.886 | 1.200 | 1.800 | 1.800 | 2.556 | 3.200 | 1.7 | 6.7 |
| **K _D_** | 0.16 | ED | <0.05 | 47 | 2.1 | 0.955 | 0.540 | 0.560 | 0.273 | <0.05 | 0.686 | 0.816 | 1.250 | 1.680 | 1.900 | 2.300 | 2.300 | 0.7 | 3.2 |
| **Na _T_** | 0.11 | ED | <0.05 | 47 | 0 | 12.134 | 55.610 | 4.580 | 0.593 | 2.000 | 3.150 | 3.500 | 4.350 | 5.140 | 8.700 | 44.600 | 385.000 | 6.6 | 44.9 |
| **Na _D_** | <0.05 | RS | 0.44 | 23 | 0 | 2.896 | 0.760 | 0.260 | 0.741 | 1.700 | 2.400 | 2.800 | 3.300 | 3.860 | 3.900 | 4.460 | 4.900 | 0.7 | 3.4 |
|  |  | DS | <0.05 | 24 | 0 | 3.613 | 1.680 | 0.470 | 0.667 | 2.600 | 2.900 | 3.150 | 3.725 | 4.480 | 4.685 | 8.102 | 11.000 | 3.7 | 16.9 |
| **Al _T_** | 0.14 | WD | <0.05 | 47 | 4.3 | 0.740 | 1.760 | 2.380 | 0.077 | <0.025 | 0.066 | 0.099 | 0.234 | 1.800 | 4.130 | 7.396 | 8.500 | 3.3 | 13.2 |
| **Al _D_** | 0.34 | WD | <0.05 | 47 | 57.5 | 0.034 | 0.040 | 1.040 | 0 | <0.025 | <0.025 | <0.025 | 0.044 | 0.071 | 0.079 | 0.109 | 0.204 | 2.8 | 13.0 |
| **Fe _T_^†^** | <0.05 | RS | <0.05 | 56 | 0 | 2.926 | 3.190 | 1.090 | 1.112 | 0.327 | 1.175 | 1.850 | 3.075 | 5.700 | 10.350 | 13.800 | 15.000 | 2.4 | 8.4 |
|  |  | DS | <0.05 | 47 | 0 | 1.642 | 1.420 | 0.870 | 0.673 | 0.200 | 0.824 | 1.200 | 2.000 | 3.680 | 4.670 | 6.032 | 6.400 | 1.8 | 6.0 |
| **Fe _D_^†^** | 0.14 | ED | <0.05 | 107 | 3.7 | 0.643 | 0.940 | 1.460 | 0.280 | <0.05 | 0.170 | 0.301 | 0.694 | 1.440 | 2.450 | 3.216 | 7.000 | 3.8 | 22.7 |
| **Mn _T_^†^** | <0.05 | RS | <0.05 | 56 | 0 | 0.347 | 0.280 | 0.820 | 0.137 | 0.071 | 0.149 | 0.223 | 0.465 | 0.761 | 0.971 | 1.100 | 1.200 | 1.5 | 4.3 |
|  |  | DS | <0.05 | 51 | 0 | 0.224 | 0.200 | 0.870 | 0.087 | 0.036 | 0.104 | 0.161 | 0.225 | 0.519 | 0.673 | 0.785 | 0.899 | 1.9 | 5.8 |
| **Mn _D_^†^** | 0.06 | ED | <0.05 | 107 | 0.9 | 0.220 | 0.220 | 1.000 | 0.114 | <0.005 | 0.084 | 0.141 | 0.282 | 0.537 | 0.750 | 0.888 | 1.000 | 1.8 | 5.7 |
| **Total phosphorus (P)^†^** | 0.58 | ED | <0.05 | 107 | 73.8 | 0.019 | 0.030 | 1.860 | 0 | <0.0075 | <0.0075 | <0.0075 | 0.014 | 0.056 | 0.098 | 0.137 | 0.190 | 2.9 | 11.4 |
| **Chloride (Cl⁻)^†^** | 0.99 | ED | <0.05 | 107 | 73.8 | 0.52 | 0.77 | 1.48 | 0 | <0.5 | <0.5 | <0.5 | 0.52 | 1.10 | 1.54 | 2.09 | 7.00 | 6.0 | 48.3 |
| **Sulfate (SO_4_^2-^)** | 0.74 | ED | <0.05 | 47 | 80.9 | 0.23 | 0.69 | 3.08 | 0 | <0.17 | <0.17 | <0.17 | <0.17 | 0.21 | 0.39 | 1.19 | 4.80 | 6.3 | 42.0 |
| **Nitrate (N-NO₃⁻)^†^** | 0.49 | ED | <0.05 | 107 | 77.6 | 0.12 | 0.29 | 2.32 | 0 | <0.11 | <0.11 | <0.11 | <0.11 | 0.23 | 0.33 | 0.62 | 2.40 | 6.2 | 45.2 |
| **Ammonia nitrogen (N-NH_3_^-^)^†^** | 0.40 | ED | <0.05 | 107 | 89.7 | 0.08 | 0.27 | 3.50 | 0 | <0.05 | <0.05 | <0.05 | <0.05 | <0.05 | 0.16 | 0.94 | 1.98 | 6.1 | 39.6 |
| **Organic nitrogen (N_org_)^†^** | 0.29 | ED | <0.05 | 107 | 72.0 | 0.52 | 0.86 | 1.65 | 0 | <0.5 | <0.5 | <0.5 | 0.55 | 0.97 | 1.51 | 1.95 | 8.22 | 7.0 | 60.8 |

## Industrial and dam area over granitic terrain (IAD-Gr)

| **Parameter** | **pMW** | **Dataset** | **pSW** | **n** | **% <LQ** | **µ** | **s** | **CV** | **MAD** | **Min** | **P25** | **P50** | **P75** | **P90** | **P95** | **P98** | **Max** | **Skewness** | **Kurtosis** |
| --- | --- | --- | --- | --- | --- | --- | --- | --- | --- | --- | --- | --- | --- | --- | --- | --- | --- | --- | --- |
| **Electrical conductivity (EC)^†^** | 0.78 | ED | 0.11 | 34 | 0 | 108.7 | 45.3 | 0.4 | 44.0 | 40.0 | 76.4 | 104.3 | 140.0 | 170.1 | 190.2 | 209.5 | 214.3 | 0.6 | 2.7 |
| **Dissolved oxygen (DO)^†^** | 0.71 | ED | <0.05 | 40 | 0 | 6.72 | 0.85 | 0.13 | 0.63 | 3.31 | 6.33 | 6.77 | 7.11 | 7.51 | 7.93 | 8.42 | 8.78 | -1.1 | 8.2 |
| **pH^†^** | 0.79 | ED | <0.05 | 40 | 0 | 7.30 | 0.42 | 0.06 | 0.36 | 6.07 | 7.15 | 7.34 | 7.62 | 7.77 | 7.81 | 7.90 | 7.90 | -1.0 | 4.0 |
| **Turbidity (TB)^†^** | 0.59 | ED | <0.05 | 40 | 0 | 35.40 | 73.57 | 2.08 | 17.96 | 1.75 | 8.10 | 20.15 | 31.18 | 45.75 | 86.54 | 216.64 | 460.00 | 5.1 | 29.3 |
| **Total alkalinity (Alk)^†^** | 0.18 | ED | <0.05 | 40 | 0 | 75.02 | 38.82 | 0.52 | 26.32 | 16.91 | 53.79 | 62.92 | 87.71 | 114.30 | 126.01 | 162.96 | 247.92 | 2.3 | 11.0 |
| **Total dissolved solids (TDS)^†^** | 0.47 | ED | <0.05 | 40 | 2.5 | 92.99 | 46.57 | 0.50 | 34.10 | <5 | 65.75 | 79.00 | 119.50 | 147.30 | 170.00 | 188.70 | 255.00 | 1.0 | 5.1 |
| **Total suspended solids (TSS)^†^** | 0.39 | ED | <0.05 | 40 | 15.0 | 38.60 | 111.88 | 2.90 | 10.38 | <5 | 6.00 | 9.50 | 21.00 | 41.20 | 106.40 | 355.98 | 675.00 | 5.0 | 28.0 |
| **Settleable solids (SS)^†^** | 0.70 | ED | <0.05 | 40 | 92.5 | <0.1 | 0.01 | 0.25 | 0 | <0.1 | <0.1 | <0.1 | <0.1 | <0.1 | 0.10 | 0.10 | 0.10 | 3.2 | 11.4 |
| **Total solids (TS)^†^** | 0.79 | ED | <0.05 | 40 | 0 | 132.15 | 125.06 | 0.95 | 37.07 | 10.00 | 80.00 | 104.00 | 146.50 | 175.00 | 279.85 | 452.88 | 807.00 | 4.2 | 22.7 |
| **Thermotolerant coliforms (TC)** | 0.60 | ED | <0.05 | 22 | 0 | 17310 | 10493 | 1 | 0 | 230 | 6100 | >16000 | >16000 | >16000 | >16000 | >16000 | >16000 | -1.0 | 2.0 |
| **Ca _T_** | 0.16 | ED | <0.05 | 23 | 0 | 14.083 | 8.210 | 0.580 | 12.750 | 3.700 | 6.450 | 15.000 | 23.000 | 25.600 | 26.000 | 26.000 | 26.000 | 0.3 | 1.5 |
| **Ca _D_** | 0.17 | ED | <0.05 | 23 | 0 | 12.060 | 7.280 | 0.600 | 10.675 | 0.784 | 5.450 | 13.000 | 16.500 | 23.000 | 23.000 | 23.560 | 24.000 | 0.3 | 1.8 |
| **Mg _T_** | 0.81 | ED | 0.13 | 23 | 0 | 4.274 | 1.060 | 0.250 | 1.483 | 2.600 | 3.250 | 4.400 | 5.050 | 5.640 | 5.880 | 6.068 | 6.200 | 0.2 | 1.8 |
| **Mg _D_** | 0.60 | ED | 0.24 | 23 | 0 | 3.896 | 0.860 | 0.220 | 1.038 | 2.500 | 3.100 | 4.000 | 4.400 | 5.160 | 5.200 | 5.256 | 5.300 | 0.1 | 1.9 |
| **K _T_** | 0.10 | ED | <0.05 | 23 | 0 | 2.496 | 2.190 | 0.880 | 0.890 | 1.100 | 1.550 | 2.000 | 2.700 | 3.280 | 3.390 | 8.216 | 12.000 | 3.8 | 17.0 |
| **K _D_** | 0.07 | ED | <0.05 | 23 | 0 | 1.747 | 0.610 | 0.350 | 0.445 | 0.972 | 1.200 | 1.700 | 1.950 | 2.860 | 2.900 | 2.956 | 3.000 | 0.8 | 2.7 |
| **Na _T_** | <0.05 | RS | 0.57 | 11 | 0 | 10.700 | 5.430 | 0.510 | 6.968 | 3.800 | 6.450 | 9.700 | 14.000 | 15.000 | 18.500 | 20.600 | 22.000 | 0.6 | 2.7 |
|  |  | DS | 0.98 | 12 | 0 | 19.333 | 7.940 | 0.410 | 8.154 | 6.000 | 13.750 | 20.000 | 24.250 | 29.500 | 31.350 | 32.340 | 33.000 | 0.1 | 2.2 |
| **Na _D_** | <0.05 | RS | 0.59 | 11 | 0 | 9.582 | 4.680 | 0.490 | 5.189 | 3.700 | 6.050 | 8.300 | 12.500 | 15.000 | 16.500 | 17.400 | 18.000 | 0.4 | 2.0 |
|  |  | DS | 0.70 | 12 | 0 | 16.425 | 6.970 | 0.420 | 8.154 | 5.200 | 11.250 | 18.500 | 20.250 | 22.800 | 25.700 | 27.680 | 29.000 | 0.0 | 2.1 |
| **Al _T_** | 0.07 | WD | <0.05 | 23 | 8.7 | 0.278 | 0.660 | 2.360 | 0.058 | <0.025 | 0.059 | 0.078 | 0.197 | 0.423 | 0.704 | 2.114 | 3.200 | 4.1 | 18.6 |
| **Al _D_** | 0.08 | WD | <0.05 | 23 | 65.2 | <0.025 | 0.020 | 0.930 | 0 | <0.025 | <0.025 | <0.025 | 0.035 | 0.046 | 0.057 | 0.087 | 0.110 | 2.5 | 9.3 |
| **Fe _T_^†^** | 0.63 | ED | <0.05 | 38 | 0 | 2.386 | 3.350 | 1.400 | 0.593 | 0.267 | 1.100 | 1.500 | 1.875 | 3.030 | 8.940 | 13.560 | 18.000 | 3.5 | 15.3 |
| **Fe _D_^†^** | 0.85 | ED | <0.05 | 40 | 17.5 | 0.270 | 0.340 | 1.260 | 0.176 | <0.05 | 0.081 | 0.202 | 0.318 | 0.525 | 0.563 | 0.903 | 2.100 | 4.0 | 22.3 |
| **Mn _T_^†^** | 0.90 | ED | <0.05 | 40 | 0 | 0.438 | 0.340 | 0.770 | 0.176 | 0.089 | 0.257 | 0.333 | 0.498 | 0.912 | 0.998 | 1.332 | 1.800 | 2.1 | 8.3 |
| **Mn _D_^†^** | 0.65 | ED | <0.05 | 40 | 0 | 0.334 | 0.290 | 0.850 | 0.173 | 0.011 | 0.162 | 0.268 | 0.387 | 0.568 | 0.691 | 1.072 | 1.700 | 3.0 | 14.3 |
| **Total phosphorus (P)^†^** | 0.16 | ED | <0.05 | 40 | 75.0 | 0.021 | 0.050 | 2.440 | 0 | <0.0075 | <0.0075 | <0.0075 | <0.0075 | 0.030 | 0.132 | 0.211 | 0.235 | 3.4 | 13.5 |
| **Chloride (Cl⁻)^†^** | 0.37 | ED | <0.05 | 40 | 5.0 | 1.63 | 0.85 | 0.52 | 0.53 | <0.5 | 1.10 | 1.35 | 1.93 | 2.82 | 3.51 | 3.69 | 4.00 | 1.1 | 3.8 |
| **Sulfate (SO_4_^2-^)** | 0.56 | ED | <0.05 | 23 | 0 | 1.58 | 0.95 | 0.60 | 0.55 | 0.38 | 1.08 | 1.30 | 1.85 | 2.76 | 3.07 | 3.94 | 4.60 | 1.6 | 5.7 |
| **Nitrate (N-NO₃⁻)^†^** | 0.85 | ED | <0.05 | 39 | 71.8 | <0.11 | 0.17 | 1.60 | 0 | <0.11 | <0.11 | <0.11 | <0.11 | 0.17 | 0.21 | 0.42 | 1.10 | 5.3 | 31.1 |
| **Ammonia nitrogen (N-NH_3_^-^)^†^** | 0.17 | ED | <0.05 | 40 | 80.0 | 0.11 | 0.35 | 3.08 | 0 | <0.05 | <0.05 | <0.05 | <0.05 | 0.23 | 0.29 | 0.85 | 2.16 | 5.5 | 32.7 |
| **Organic nitrogen (N_org_)^†^** | 0.88 | ED | <0.05 | 40 | 82.5 | <0.5 | 0.15 | 0.47 | 0 | <0.5 | <0.5 | <0.5 | <0.5 | 0.56 | 0.65 | 0.72 | 0.73 | 1.9 | 5.1 |

## Urban influence over mixed geology (UI-Mixed)

| **Parameter** | **pMW** | **Dataset** | **pSW** | **n** | **% <LQ** | **µ** | **s** | **CV** | **MAD** | **Min** | **P25** | **P50** | **P75** | **P90** | **P95** | **P98** | **Max** | **Skewness** | **Kurtosis** |
| --- | --- | --- | --- | --- | --- | --- | --- | --- | --- | --- | --- | --- | --- | --- | --- | --- | --- | --- | --- |
| **Electrical conductivity (EC)^†^** | <0.05 | RS | 0.08 | 64 | 0 | 100.6 | 53.5 | 0.5 | 54.4 | 12.2 | 65.9 | 101.4 | 137.2 | 161.8 | 177.3 | 218.4 | 238.2 | 0.2 | 2.7 |
|  |  | DS | <0.05 | 54 | 0 | 145.34 | 93.18 | 0.64 | 117.94 | 15.00 | 73.58 | 142.00 | 221.83 | 291.00 | 304.18 | 308.63 | 319.00 | 0.3 | 1.9 |
| **Dissolved oxygen (DO)^†^** | 0.71 | ED | <0.05 | 192 | 0 | 6.61 | 0.65 | 0.10 | 0.65 | 3.54 | 6.15 | 6.51 | 7.13 | 7.38 | 7.58 | 7.81 | 8.14 | -0.5 | 4.8 |
| **pH^†^** | 0.39 | ED | <0.05 | 192 | 0 | 7.22 | 0.39 | 0.05 | 0.32 | 5.69 | 7.05 | 7.28 | 7.46 | 7.67 | 7.73 | 7.87 | 8.40 | -0.8 | 4.4 |
| **Turbidity (TB)^†^** | <0.05 | RS | <0.05 | 102 | 0 | 124.40 | 250.46 | 2.01 | 43.66 | 1.70 | 12.48 | 34.95 | 98.90 | 322.50 | 509.31 | 1094.14 | 1530.00 | 3.7 | 18.1 |
|  |  | DS | <0.05 | 90 | 0 | 18.52 | 27.07 | 1.46 | 9.86 | 0.37 | 5.33 | 12.05 | 18.43 | 31.03 | 54.08 | 130.32 | 140.00 | 3.4 | 14.8 |
| **Total alkalinity (Alk)^†^** | <0.05 | RS | <0.05 | 102 | 2.0 | 30.97 | 15.23 | 0.49 | 9.27 | <6 | 14.16 | 36.45 | 40.00 | 45.30 | 48.67 | 56.12 | 69.83 | -0.4 | 2.3 |
|  |  | DS | <0.05 | 89 | 1.1 | 37.80 | 20.49 | 0.54 | 27.37 | <6 | 16.50 | 37.79 | 53.67 | 62.00 | 64.35 | 72.00 | 86.67 | 0.0 | 2.1 |
| **Total dissolved solids (TDS)^†^** | <0.05 | RS | <0.05 | 102 | 0 | 75.55 | 39.38 | 0.52 | 35.58 | 10.00 | 49.25 | 86.00 | 102.75 | 117.90 | 126.75 | 137.00 | 158.00 | -0.4 | 2.1 |
|  |  | DS | <0.05 | 89 | 2.3 | 101.57 | 72.28 | 0.71 | 96.37 | <5 | 28.00 | 110.00 | 158.00 | 210.20 | 227.00 | 236.56 | 255.00 | 0.2 | 2.0 |
| **Total suspended solids (TSS)^†^** | <0.05 | RS | <0.05 | 102 | 15.7 | 122.08 | 219.41 | 1.80 | 49.67 | <5 | 11.00 | 36.00 | 101.50 | 358.10 | 609.10 | 697.18 | 1313.00 | 3.3 | 14.9 |
|  |  | DS | <0.05 | 89 | 23.6 | 31.97 | 69.64 | 2.18 | 13.34 | <5 | 5.00 | 14.00 | 23.00 | 45.60 | 110.40 | 274.96 | 457.00 | 4.4 | 23.3 |
| **Settleable solids (SS)^†^** | <0.05 | RS | <0.05 | 102 | 60.8 | 0.50 | 2.21 | 4.46 | 0 | <0.1 | <0.1 | <0.1 | 0.20 | 1.00 | 1.98 | 2.00 | 22.00 | 9.2 | 90.0 |
|  |  | DS | <0.05 | 90 | 76.7 | 0.11 | 0.17 | 1.58 | 0 | <0.1 | <0.1 | <0.1 | <0.1 | 0.21 | 0.46 | 0.61 | 1.00 | 4.0 | 19.8 |
| **Total solids (TS)^†^** | 0.29 | ED | <0.05 | 191 | 0 | 168.64 | 184.27 | 1.09 | 99.33 | 5.00 | 49.50 | 139.00 | 190.00 | 294.00 | 513.50 | 728.80 | 1404.00 | 3.4 | 19.0 |
| **Thermotolerant coliforms (TC)** | <0.05 | RS | <0.05 | 37 | 0 | 19599 | 8650 | 0 | 0 | 45 | >16000 | >16000 | >16000 | >16000 | >16000 | >16000 | >16000 | -1.6 | 3.9 |
|  |  | DS | <0.05 | 40 | 5.0 | 13609 | 11560 | 1 | 0 | <18 | 330 | >16000 | >16000 | >16000 | >16000 | >16000 | >16000 | -0.3 | 1.1 |
| **Ca _T_** | 0.71 | ED | <0.05 | 79 | 0 | 9.897 | 5.300 | 0.540 | 3.855 | 0.779 | 7.700 | 11.000 | 13.000 | 15.000 | 16.100 | 21.080 | 26.000 | 0.1 | 3.5 |
| **Ca _D_** | 0.92 | ED | <0.05 | 79 | 0 | 8.291 | 4.060 | 0.490 | 2.817 | 0.581 | 6.300 | 9.100 | 11.000 | 13.000 | 14.000 | 14.000 | 14.000 | -0.6 | 2.3 |
| **Mg _T_** | 0.11 | ED | <0.05 | 79 | 0 | 2.921 | 1.020 | 0.350 | 0.593 | 0.688 | 2.650 | 3.200 | 3.500 | 3.920 | 4.210 | 4.476 | 4.700 | -0.8 | 2.8 |
| **Mg _D_** | <0.05 | RS | <0.05 | 39 | 0 | 2.355 | 0.790 | 0.340 | 0.593 | 0.630 | 2.050 | 2.600 | 2.900 | 3.220 | 3.400 | 3.424 | 3.500 | -0.8 | 2.5 |
|  |  | DS | <0.05 | 40 | 0 | 2.684 | 0.930 | 0.350 | 0.741 | 0.708 | 2.300 | 2.950 | 3.300 | 3.500 | 3.815 | 4.166 | 4.400 | -0.7 | 2.7 |
| **K _T_** | <0.05 | RS | <0.05 | 39 | 0 | 2.223 | 1.020 | 0.460 | 0.593 | 0.317 | 1.900 | 2.400 | 2.800 | 3.400 | 3.920 | 4.100 | 4.100 | -0.4 | 2.7 |
|  |  | DS | 0.17 | 40 | 0 | 3.413 | 1.980 | 0.580 | 2.224 | 0.171 | 2.500 | 3.100 | 4.825 | 5.400 | 6.250 | 7.574 | 8.900 | 0.3 | 3.1 |
| **K _D_** | <0.05 | RS | 0.06 | 39 | 0 | 1.942 | 0.920 | 0.470 | 0.741 | 0.150 | 1.700 | 1.900 | 2.550 | 2.880 | 3.220 | 3.568 | 4.100 | -0.3 | 2.9 |
|  |  | DS | <0.05 | 40 | 0 | 2.782 | 1.520 | 0.550 | 1.779 | 0.108 | 2.050 | 2.750 | 4.025 | 4.810 | 4.905 | 5.066 | 5.300 | -0.3 | 2.1 |
| **Na _T_** | <0.05 | RS | 0.07 | 39 | 5.1 | 8.254 | 5.200 | 0.630 | 5.782 | <0.5 | 4.350 | 8.500 | 12.000 | 16.000 | 17.000 | 17.240 | 18.000 | 0.3 | 2.0 |
|  |  | DS | 0.07 | 40 | 5.0 | 17.428 | 10.840 | 0.620 | 11.120 | <0.5 | 8.175 | 19.000 | 26.000 | 31.200 | 34.000 | 35.100 | 39.000 | -0.1 | 2.1 |
| **Na _D_** | <0.05 | RS | 0.08 | 39 | 7.7 | 6.993 | 4.750 | 0.680 | 4.003 | <0.5 | 3.900 | 5.800 | 10.400 | 14.000 | 14.100 | 15.720 | 18.000 | 0.4 | 2.2 |
|  |  | DS | 0.08 | 40 | 10.0 | 14.834 | 10.120 | 0.680 | 10.378 | <0.5 | 6.225 | 15.500 | 22.000 | 26.300 | 32.050 | 33.660 | 36.000 | 0.1 | 2.1 |
|  |  | DS | <0.05 | 39 | 64.1 | 0.026 | 0.020 | 0.870 | 0 | <0.025 | <0.025 | <0.025 | 0.033 | 0.056 | 0.069 | 0.094 | 0.101 | 1.8 | 5.8 |
| **Al _T_** | <0.05 | RS | <0.05 | 39 | 0 | 2.324 | 4.520 | 1.940 | 0.424 | 0.044 | 0.155 | 0.338 | 1.950 | 7.580 | 10.700 | 17.720 | 20.000 | 2.7 | 9.9 |
|  |  | DS | <0.05 | 40 | 7.5 | 0.481 | 1.400 | 2.920 | 0.067 | <0.025 | 0.094 | 0.147 | 0.186 | 0.381 | 1.136 | 6.086 | 7.100 | 4.1 | 18.4 |
| **Al _D_** | <0.05 | RS | <0.05 | 39 | 28.2 | 0.063 | 0.070 | 1.080 | 0.050 | <0.025 | <0.025 | 0.046 | 0.079 | 0.153 | 0.165 | 0.221 | 0.375 | 2.8 | 12.6 |
|  |  | DS | <0.05 | 39 | 64.1 | 0.026 | 0.020 | 0.870 | 0 | <0.025 | <0.025 | <0.025 | 0.033 | 0.056 | 0.069 | 0.094 | 0.101 | 1.8 | 5.8 |
| **Fe _T_^†^** | <0.05 | RS | <0.05 | 102 | 0 | 3.556 | 5.460 | 1.540 | 1.317 | 0.136 | 0.834 | 1.400 | 3.825 | 8.950 | 13.950 | 21.980 | 30.000 | 3.1 | 13.1 |
|  |  | DS | <0.05 | 84 | 0 | 1.371 | 2.640 | 1.920 | 0.195 | 0.083 | 0.716 | 0.869 | 1.000 | 1.270 | 2.555 | 8.852 | 20.000 | 5.7 | 37.0 |
| **Fe _D_^†^** | <0.05 | RS | <0.05 | 102 | 12.8 | 0.223 | 0.170 | 0.740 | 0.139 | <0.05 | 0.113 | 0.195 | 0.292 | 0.421 | 0.564 | 0.668 | 0.907 | 1.4 | 5.7 |
|  |  | DS | <0.05 | 89 | 12.4 | 0.258 | 0.150 | 0.590 | 0.144 | <0.05 | 0.156 | 0.262 | 0.353 | 0.431 | 0.552 | 0.596 | 0.691 | 0.4 | 3.0 |
| **Mn _T_^†^** | 0.54 | ED | <0.05 | 191 | 0 | 0.953 | 2.020 | 2.120 | 0.377 | 0.063 | 0.389 | 0.607 | 0.909 | 1.500 | 2.450 | 4.800 | 26.000 | 10.2 | 124.7 |
| **Mn _D_^†^** | <0.05 | RS | <0.05 | 102 | 0 | 0.303 | 0.210 | 0.690 | 0.162 | 0.008 | 0.149 | 0.249 | 0.374 | 0.588 | 0.729 | 0.849 | 0.938 | 1.1 | 3.7 |
|  |  | DS | <0.05 | 89 | 0 | 0.538 | 0.300 | 0.550 | 0.279 | 0.048 | 0.344 | 0.560 | 0.713 | 0.991 | 1.060 | 1.100 | 1.100 | 0.0 | 2.2 |
| **Total phosphorus (P)^†^** | <0.05 | RS | <0.05 | 102 | 36.3 | 0.077 | 0.080 | 1.040 | 0.098 | <0.0075 | <0.0075 | 0.070 | 0.114 | 0.180 | 0.260 | 0.306 | 0.317 | 1.2 | 4.2 |
|  |  | DS | <0.05 | 88 | 26.1 | 0.137 | 0.120 | 0.840 | 0.159 | <0.0075 | <0.0075 | 0.143 | 0.208 | 0.298 | 0.342 | 0.377 | 0.381 | 0.4 | 2.0 |
| **Chloride (Cl⁻)^†^** | <0.05 | RS | <0.05 | 102 | 25.5 | 3.77 | 2.92 | 0.77 | 3.93 | <0.5 | <0.5 | 3.60 | 5.88 | 7.38 | 9.37 | 9.50 | 12.00 | 0.5 | 2.6 |
|  |  | DS | <0.05 | 89 | 23.6 | 8.46 | 6.46 | 0.76 | 8.30 | <0.5 | 0.89 | 9.40 | 13.00 | 16.20 | 20.00 | 20.24 | 22.00 | 0.1 | 1.9 |
| **Sulfate (SO_4_^2-^)** | <0.05 | RS | <0.05 | 39 | 10.3 | 3.43 | 2.36 | 0.69 | 2.67 | <0.17 | 2.00 | 3.00 | 4.95 | 6.54 | 7.61 | 7.77 | 8.00 | 0.3 | 2.1 |
|  |  | DS | <0.05 | 39 | 20.5 | 7.14 | 4.62 | 0.65 | 5.19 | <0.17 | 3.75 | 7.50 | 11.00 | 12.20 | 13.10 | 14.24 | 15.00 | -0.3 | 1.9 |
| **Nitrate (N-NO₃⁻)^†^** | <0.05 | RS | <0.05 | 100 | 30.0 | 0.73 | 0.73 | 1.01 | 0.89 | <0.11 | <0.11 | 0.70 | 1.20 | 1.50 | 1.60 | 1.81 | 5.20 | 2.4 | 14.9 |
|  |  | DS | <0.05 | 87 | 21.8 | 1.39 | 1.11 | 0.80 | 1.07 | <0.11 | 0.11 | 1.60 | 2.10 | 2.44 | 2.91 | 3.68 | 5.90 | 0.7 | 4.5 |
| **Ammonia nitrogen (N-NH_3_^-^)^†^** | <0.05 | RS | <0.05 | 102 | 52.9 | 0.22 | 0.41 | 1.88 | 0 | <0.05 | <0.05 | <0.05 | 0.19 | 0.65 | 1.16 | 1.54 | 2.21 | 3.2 | 13.7 |
|  |  | DS | <0.05 | 89 | 36.0 | 0.68 | 0.93 | 1.38 | 0.35 | <0.05 | <0.05 | 0.26 | 1.05 | 1.81 | 2.11 | 3.69 | 4.78 | 2.2 | 8.6 |
| **Organic nitrogen (N_org_)^†^** | 0.78 | ED | <0.05 | 191 | 39.3 | 2.66 | 3.38 | 1.27 | 1.57 | <0.5 | <0.5 | 1.31 | 4.10 | 6.79 | 9.79 | 12.20 | 18.00 | 1.9 | 6.9 |

# Appendix B – Spatial variations of the Congonhas Mineral District

Appendix B illustrates the spatial variation observed in the study area, emphasizing the influence of geology on relief and land-use patterns. In the northwestern portion, relatively better-preserved mountainous terrain (BPA-MVS; Fig. B1a, b) and areas under mining influence (MA-MVS; Fig. B1c), both developed over metavolcano-sedimentary rocks, where lithotypes of geochemical and economic relevance, such as manganiferous formations (Fig. B1d) and banded iron formations (Fig. B1e) outcrop, putting in evidence the strong metalliferous signature that is characteristic of northern part of the study area. In contrast, the southern portion is marked by undulating relief associated with granitoid rocks, with a significant presence of pasturelands and other uses (Fig. B1f, g). This compartmentalization highlights the close relationship between the geological substrate, morphology, and land use, which are fundamental aspects for understanding the spatial variations in surface water geochemistry and environmental processes in the Congonhas Mineral District region.


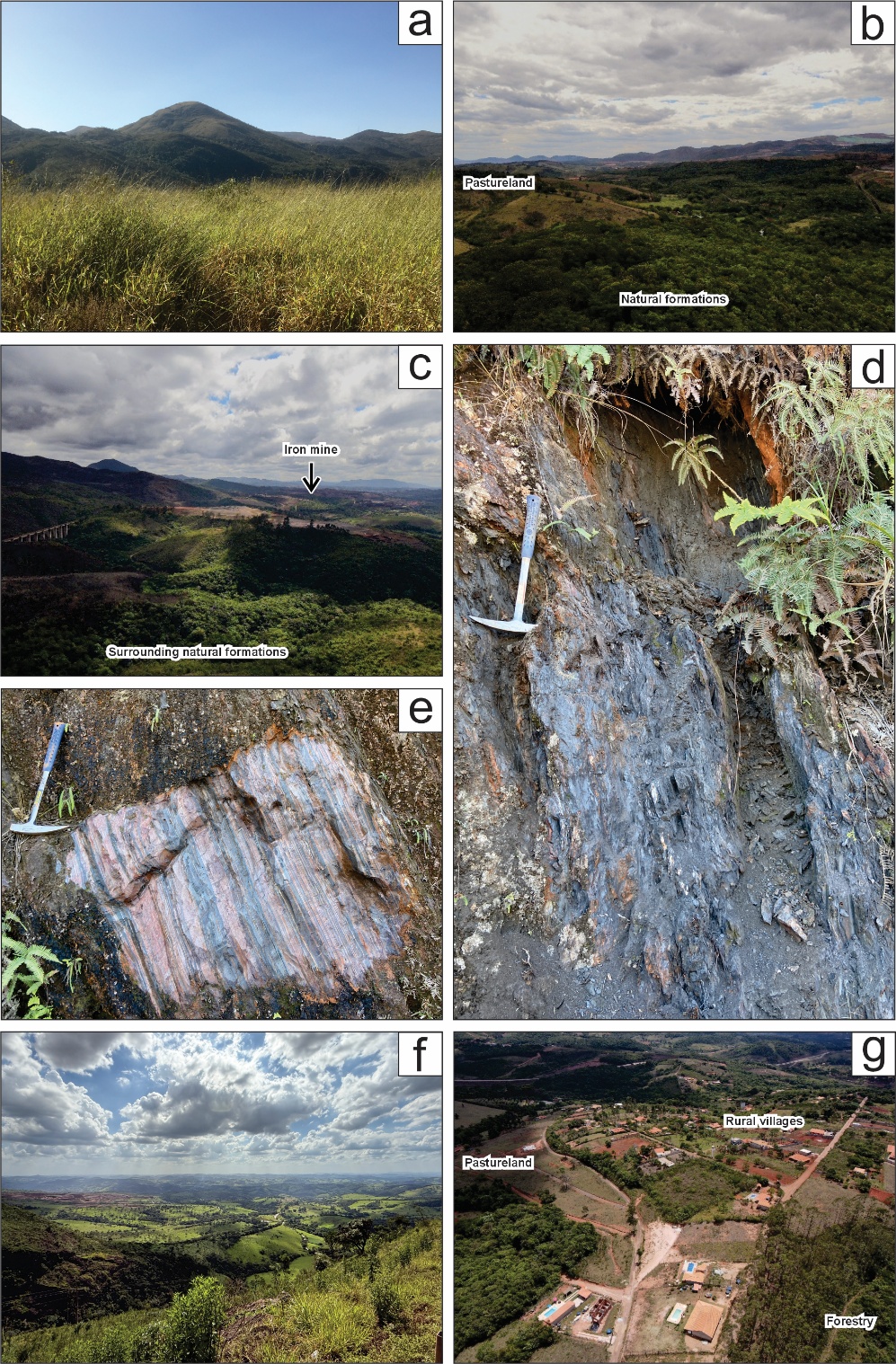


Figure B1 Spatial variation in the study area. Relatively better-preserved mountainous terrain (a-b) and mining area (c) developed over metavolcano-sedimentary rocks in the northwestern part of the study area, with occurrences of manganiferous rocks (d) and banded iron formations (e). Undulating terrain developed over granitoids (f) with presence of multiple land uses, particularly pastureland, in the southern portion of the study area (g).

# Appendix C – Spatial distribution maps of water quality indicators

Spatial distribution maps of dissolved oxygen, pH, ammonia nitrogen, organic nitrogen, thermotolerant coliforms, turbidity, total dissolved solids, settleable solids, total suspended solids, total solids, based on the median values from the historical series covering two hydrological cycles of selected water quality indicators during the rainy and dry seasons in the study area. The locations of the main Fe and Mn mines in the region are indicated on the maps, along with the abbreviations: C = city of Congonhas-MG, I = industrial area, and D = Fe mining tailings dam. The boxplots represent all measurements from the sampling sites grouped according to their respective geospatial class (see Fig. 2): BPA-MVS: Better-preserved areas over metavolcano-sedimentary rocks; MA-MVS: Mining areas over metavolcano-sedimentary rocks; MU-Gr: Mixed land uses (pastureland) over granitic terrain; IAD-Gr: Industrial area and tailings over granitic terrain; UI-Mixed: Urban influence over mixed geology. The red dashed line indicates the Class 2 freshwater regulatory limits established by the Brazilian National Environment Council (CONAMA, 2005).


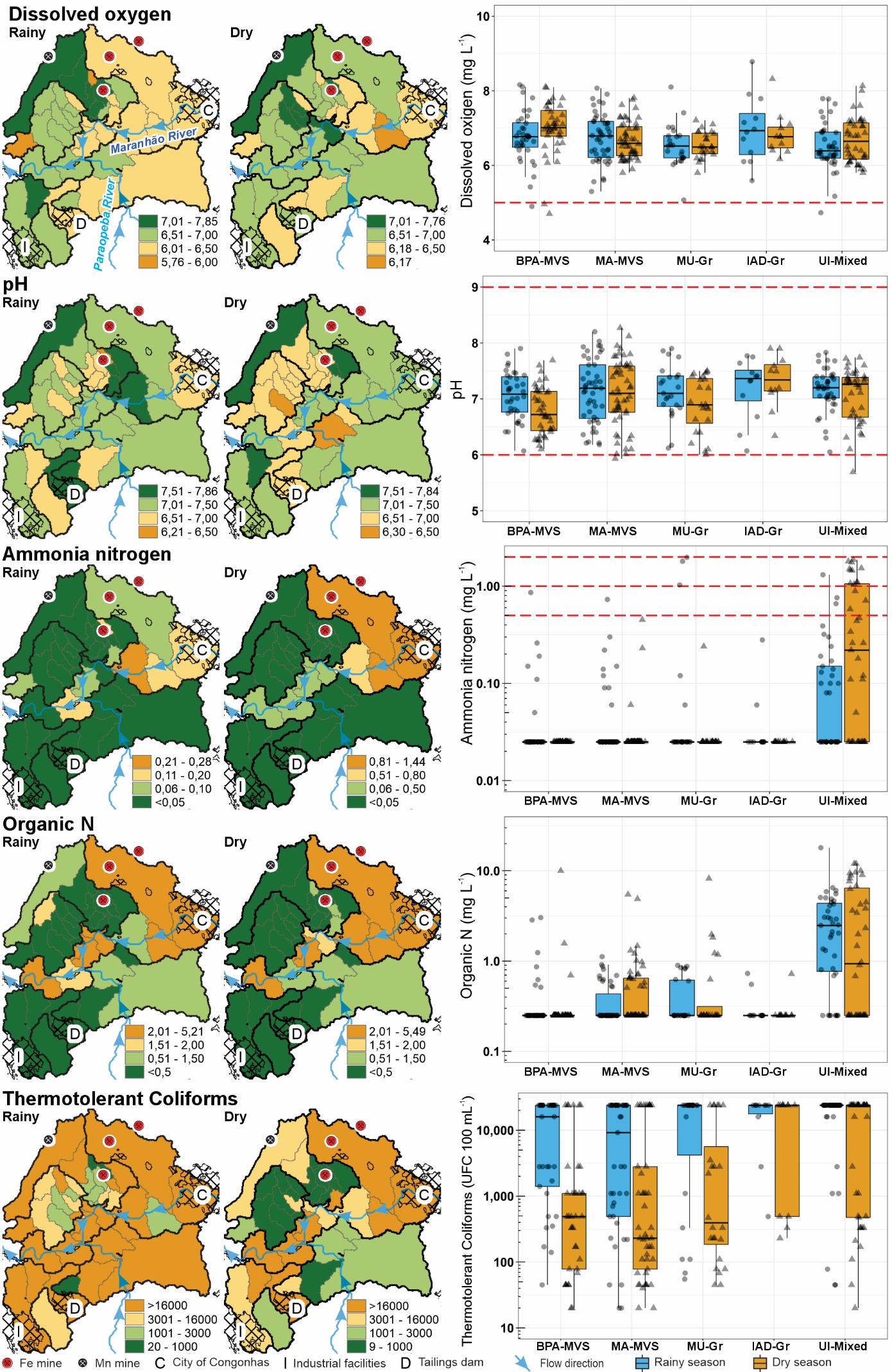


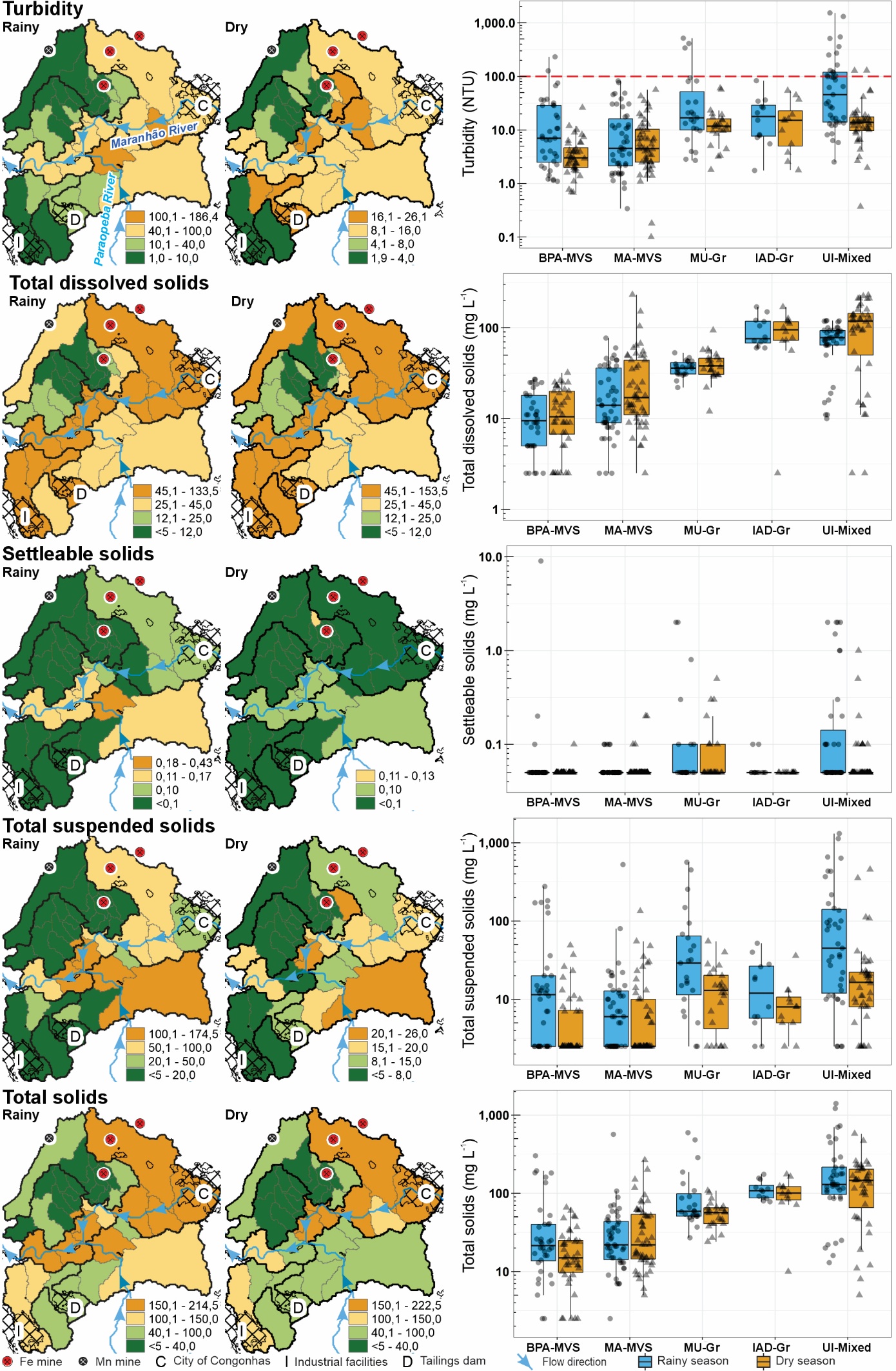

Supplement: Supplementary file 1 — Supplementary file1 (DOCX 3002 kb) [file 10653_2026_2989_MOESM1_ESM.docx]
